# Geographical-Scale Evidence Reveals Plant Nutrient as an Effective Indicator for Coastal Carbon Emissions

**DOI:** 10.3390/plants14182852

**Published:** 2025-09-12

**Authors:** Jing Xiong, Xuexin Shao, Haidong Xu, Ming Wu

**Affiliations:** 1 Research Institute of Subtropical Forestry, Chinese Academy of Forestry, Hangzhou 311400, China; xiongxiaojing2016@163.com; 2 School of Life Sciences, Jinggangshan University, Ji’an 343009, China; 3 Wetland Ecosystem Research Station of Hangzhou Bay, Research Institute of Subtropical Forestry, Chinese Academy of Forestry, Hangzhou 311400, China; 4 State Key Laboratory of Wetland Conservation and Restoration, Beijing 100080, China; 5 Yellow River Delta Ecological Environment Research Center, Shandong University of Aeronautics, Binzhou 256603, China; haidongxuu@163.com

**Keywords:** methane, carbon dioxide, plant trait, coastal wetland, geographical-scale

## Abstract

Plant traits could help in designing feasible strategies to mitigate global change in inland wetlands, but the correlations between plant traits and carbon emissions in coastal wetlands remain unclear. Here, we investigated the plant traits (including nutrient, structural, and biomass traits) and environmental conditions (including climate and soil properties) and determined the soil carbon emissions (methane (CH_4_), carbon dioxide (CO_2_), and their temperature sensitivities (*Q*_10_ value)) from the soils of 90 coastal herbaceous wetlands differing in land use types along China’s coastline. We further tested how environmental conditions affected plant traits and how these traits then altered carbon emissions. We found that plant traits had a greater effect on CH_4_ and CO_2_ emissions than on their *Q*_10_ values, with nutrient traits being the key drivers in coastal herbaceous wetlands in China. In general, coastal herbaceous wetlands with larger leaf C and N contents combined with a lower leaf N:P ratio tended to have higher CH_4_ emission; those with larger leaf C and P contents combined with a lower leaf N:P ratio tended to have higher CO_2_ emission; and those with higher leaf N content and N:P ratio combined with a lower leaf C:P ratio tended to have higher *Q*_10_ values of both CH_4_ and CO_2_. Notably, the predictive power of plant traits in coastal herbaceous wetlands varied significantly across heterogeneous environments influenced by climate and land use. Our results highlight the critical role of plant nutrient traits in driving soil carbon emissions and provide practical insights into understanding coastal carbon dynamics under pressures from climate and land use changes (e.g., coastal reclamation and plant invasion).

## 1. Introduction

Climate warming is one of the global challenges threatening biodiversity, ecosystem stability, and the sustainable development of humanity. Soil is considered a crucial “nature-based solution” for climate adaption and mitigation and contains the largest proportion of the Earth’s terrestrial carbon [1]. Coastal wetlands could sequester at least 10 times more carbon than terrestrial ecosystems and hold approximately 10% of global soil carbon [2]. Meanwhile, soils contribute approximately 5–20% of atmospheric carbon dioxide (CO_2_) and 15–30% of methane (CH_4_) globally [3], which are the two largest greenhouse gases driving climate warming and the principal gaseous end products of organic carbon decomposition [4]. Particularly, the predominantly anaerobic conditions in wetland soils favor CH_4_ production [5], which exhibits a global warming potential at least 28 times greater than that of CO_2_ over a 100-year period, and can shift coastal ecosystems from net carbon sinks to net carbon sources [6].

In turn, climate warming has become a major driving factor of CO_2_ and CH_4_ emissions in wetland soils [7,8]. Most studies have revealed that rising temperatures can stimulate soil microbial respiration, potentially forming positive climate warming feedback [9,10]. However, it is also reported that soil respiration displayed temperature sensitivity (*Q*_10_) to temperature changes, which suggested that the stimulation effect of climate warming on soil respiration may be weaker than currently predicted [11,12]. Quantifying the *Q*_10_ value of soil respiration is thus essential for predicting wetland carbon–climate feedback [8,11]. However, these critical uncertainties in the direction and magnitude of carbon–climate feedback still remain, owing to a poor understanding of the *Q*_10_ values of soil respiration in coastal ecosystems.

Notably, the changes in land use are more likely to have huge uncertain feedback effects on the overall benefit of soil carbon sequestration in various ecosystems and global climate warming [13,14,15]. Globally, land use types of coastal ecosystems have been subjected to a series of human activities, particularly reclamation and exotic vegetation introduction [5,16]. Since 1970s, approximately 60% of China’s coastline length has been reclaimed [17], and 48.3% of salt marsh vegetation area has been occupied by *Spartina alterniflora* in China [18], which was reported to increase, decrease, or have a non-significant effect on CH_4_ and CO_2_ emissions from soil in different reclaimed, restored, or invasive ecosystems [5,13,19]. However, geographical-scale assessments of CH_4_ and CO_2_ emissions and their *Q*_10_ values across diverse coastal ecosystems under varying environmental conditions remain unclear.

Plant species are important for carbon emissions in wetland soils as they affect electron donors (e.g., organic carbon) and electron acceptors (e.g., *O*_2_, *Fe*^2+^, *Fe*^3+^) [20] or support an oxidizing environment in the rhizosphere to regulate methanogenesis and methanotrophy [21]. Plant traits could provide a quantitative approach to understand and predict ecosystem properties, especially for soil biogeochemical processes, which in turn could help in designing feasible strategies for global change [20,21,22]. The “response–effect” trait framework links environment, plant, and ecosystem processes and functions by quantifying trait-mediated environmental responses and their functional consequences [23,24,25,26]. “Response traits” can describe the trade-offs between resources acquisitive and conservative strategies and can be measured with traits (e.g., leaf nutrient content, leaf dry matter content (LDMC), and leaf specific area (SLA)) [27] or describe resource allocation to light competition, photosynthesis, and reproduction, which be measured with traits (e.g., primary productivity and height) [23]. “Effect traits” are those traits that affect ecosystem processes and functions [26]. In fact, the “response–effect” trait framework provides a quantitative and globally applicable approach to understand and predict plant-mediated processes of soil carbon emissions in wetland ecosystems, which in turn could help in designing mitigation strategies for climate warming. For example, in graminoid-dominated ecosystems, variation in soil CH_4_ and CO_2_ production has been linked to SLA, LDMC in tundra [21], height, leaf area, and leaf N content in peatlands [22,28], as well as plant productivity in freshwater wetlands [20]. Although coastal wetlands play a critical role in the global carbon budget, the contribution of plant traits to carbon emissions of coastal soil still remains uncertain.

Here, we evaluate the “response–effect” trait framework within the coastal wetlands in southeast China. We investigated plant traits from 90 typical coastal herbaceous wetlands of southeast China, spanning the tropics, subtropics, and temperate zones, and including natural wetlands, invasive wetlands, and reclaimed wetlands. We also collected soil samples from these coastal herbaceous wetlands and then incubated them at three temperature levels (15, 25, and 30 °C) to determine CH_4_ and CO_2_ emissions and calculate their *Q*_10_ values. The *Q*_10_ value indicates the carbon emission rate increase with each 10 °C rise in temperature, which could reflect the magnitude of the feedback between carbon emissions and climate changes [12]. Our aims are to (i) investigate the relationships between plant traits and soil carbon emissions; (ii) explore the key plant traits that predict coastal carbon emissions; and (iii) identify whether the response and effect traits are correlated in coastal herbaceous wetlands under reclaimed and invasive pressures. We hypothesized that (i) plant traits associated with soil carbon emissions exhibit significant variations across different climate and land use types; and (ii) the strength of the relationships between plant traits and soil carbon emissions is decoupled from that in coastal herbaceous wetlands compared with that in freshwater wetlands, attributed to the more extreme water, salinity, and nutrient conditions in soil ecosystems [29,30].

## 2. Materials and Methods

### 2.1. Site Description

**Figure 1:**
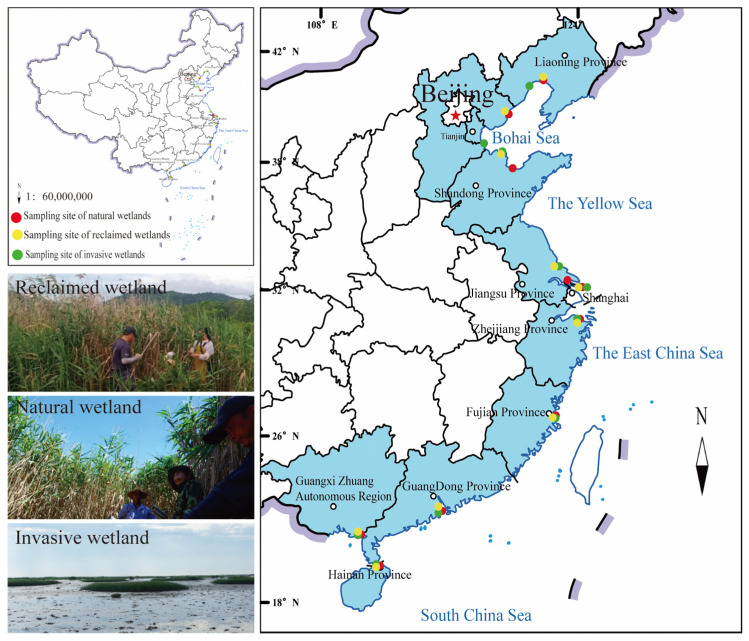
Location of the study area in Chinese coastal wetlands along the latitudinal gradient.

Ninety coastal herbaceous wetlands (3 wetland types × 10 sampling sites × 3 sampling plots) were selected and divided into three land use types, extending for 1800 km in 9 coastal provinces, spanning tropics, subtropics, and temperate climates (19.96–41.01° N, 109.91–121.99° E) (Figure 1 and Table S1). The mean annual temperature (MAT) ranges from 9.8 °C to 23.5 °C and the mean annual precipitation (MAP) ranges from 528 mm to 2082 mm. MAT and MAP were calculated based on 30-year averaged records (1981–2010) from 18 meteorological stations within or around the study area (http://data.cma.cn/) (accessed on 1 August 2020) (Table S1). The three selected coastal land uses include natural wetlands and reclaimed wetlands primarily dominated by native *Phragmites australis* and invasive wetlands principally occupied by exotic *S. alterniflora*. The sampling sites of natural wetlands are located on mid-to-high intertidal transition, those of invasive wetlands are located on low intertidal transition, and those of reclaimed wetlands are no longer affected by tidal effects or human management. For other detailed information about the sampling sites, please refer to Xiong et al. [29,30].

### 2.2. Sampling and Measurement

Vegetation sampling was conducted during the plant growing season (July to September in 2020). A randomly selected 1 m × 1 m sampling site was established at each coastal herbaceous wetland, and the total fresh aboveground biomass and density of the vegetation community were measured. Twelve plant individuals of each sampling plot were randomly selected to determine the fresh aboveground biomass, height, and diameter through the use of a handheld electronic scale, vernier caliper, and steel tape measure, respectively. The dry aboveground biomass of these 12 plants was measured after drying to constant weight. Moreover, the total dry aboveground biomass (AGB) of the vegetation community was calculated with Equation (1). In addition, thirty mature and healthy leaves from the middle and upper stem sections were randomly collected, then soaked in sterile water for 12 h, blotted dry with filter paper, and weighed to measure leaf fresh biomass. Leaf area was measured using a handheld leaf area scanner. The scanned leaves were put into an envelope and dried to constant weight, and their leaf dry biomass was measured. The specific leaf area (SLA) and leaf dry matter content (LDMC) were calculated with Equation (2) and Equation (3), respectively. Moreover, the leaves dried at 75 °C were ground and passed through a 2 mm sieve to determine leaf C, N, and P contents [31]. Specifically, the leaf C and N contents were determined using the elemental analyzer [31], and leaf P content was determined using a digestion procedure with HNO_3_–H_2_SO_4_–HClO_4_ [32]. All the measured plant traits are presented in Table 1.

(1) $$AGB=\left(\frac{Dry\;aboveground\;biomass}{Fresh\;aboveground\;biomass}\right)*Total\;fresh\;aboveground\;biomass$$

(2) $$SLA=\frac{Leaf\;area}{Leaf\;dry\;biomass}$$

(3) $$LDMC=\frac{Leaf\;fresh\;biomass}{Leaf\;dry\;biomass}$$

At each sampling site, five pits were dug to collect undisturbed soil cores (length and diameter: 100 cm and 50 mm) from the depth of 0–100 cm and divided into four depths (0–10 cm, 10–30 cm, 30–60 cm, and 60–100 cm) considering root spatial distribution and soil oxygen status [33]. These five undisturbed soil samples of each soil depth of each sampling site were combined to form a composite sample, respectively. A total of 360 soil samples were collected (3 wetland types × 10 sampling sites × 3 sampling plots × 4 soil depths). These soil samples were transported in specialized containers, subsequently sieved through a 2 mm mesh after removing visible vegetation residues, and then divided for determining incubation experiments [33].

Undisturbed soil cores (50 mm in diameter) were collected from 0–10 cm, 10–30 cm, 30–60 cm, and 60–100 cm soil depths using a soil auger, and these soil cores were then weighed to determine soil bulk density and soil moisture [34], while pH and salinity were analyzed in a 1:5 (*w*/*v*) soil–water suspension with a multifunctional pH meter and conductivity meter, respectively [35]. Total soil sulfate concentration was measured using barium sulfate turbidimetry combined with volumetric analysis [10]. Total nitrogen (N) content of soil was determined using the elemental analyzer, and the phosphorus (P) content of soil was analyzed with the molybdenum-antimony-spectrophotometric method after HNO_3_–HF–HClO_4_ digestion [31]. Dissolved carbon content (DOC) of soil was measured through 1:5 (*w*/*v*) soil–water extraction using the TOC analyzer [35]. All the measured soil properties are presented in Table 1. The distributions of these soil properties among different soil depths and land uses are exhibited in Table S2, and their latitudinal patterns are displayed in Figure S1.

### 2.3. Incubation Experiments

Three incubation temperature levels (15 °C, 25 °C, and 30 °C) were selected according to the growing season air temperature range in our study area. The coastal wetland under prolonged flooding was dominated by anaerobic conditions and then formed an excellent site for CH_4_ emission [19]. In anaerobic microbial processes, a consortium of organisms mediates organic matter decomposition into CO_2_ and CH_4_ through methanogenesis, iron reduction, and sulfate reduction [20,36]. Therefore, both CH_4_ and CO_2_ emissions in anaerobic conditions were measured by the laboratory incubation experiment.

The method for anaerobic incubation and the methods for measuring CH_4_ and CO_2_ emissions were referenced from Xiong et al. [37] and Yuan et al. [19]. Specifically, fresh soil equivalent to 10 g dry weight was weighed into a 150 mL jar and mixed with artificial seawater to form a 2.5:1 (*w*/*v*) soil–water suspension. The jars were nitrogen-flushed (>5 times) to establish anaerobic conditions, then incubated in darkness at 15 °C, 25 °C, and 30 °C, and sampled daily via 5 mL micro syringe gas extraction from headspace until stable concentrations were achieved (8 days). CH_4_ and CO_2_ emissions were measured by analyzing the rate of gas concentration change over time in sealed flasks. The CH_4_ and CO_2_ emissions were analyzed using a gas chromatograph with a flame ionization detector, and their emission rate was determined with Equation (4):

(4) $$Emission\;rate=\frac{\mathrm{dc}}{\mathrm{dt}}\cdot \frac{V_{H}}{W_{S}}\cdot \frac{MW}{MV}\cdot \frac{T_{\mathrm{st}}}{\left(T_{\mathrm{st}}+T\right)}$$

where *Emission rate* was the CH_4_ and CO_2_ emissions (μg·g^−1^·d^−1^), *dc*/*dt* was the change in the rate of CH_4_ emission or CO_2_ emission per unit time, *V_H_* was the headspace volume (L) of the culture bottle, *W_S_* was dry soil weight (g), *MW* was the molecular weight of methane or carbon dioxide (g·mol^−1^), *MV* represents the volume of 1 mole of gas under standard conditions (L), *T* was incubation temperature (°C), and *T_st_* was standard temperature (K).

The *Q*_10_ values of CH_4_ or CO_2_ emission at 15–25, 25–30, or 15–30 °C were calculated with Equation (2) [7]:

(5) $$Q_{10}={\frac{{Emission\;rate}_{T1}}{{Emission\;rate}_{T2}}\;}^{\frac{10}{T2-T1}}$$

where *Emission rate* was the CH_4_ or CO_2_ emission (μg·g^−1^·d^−1^) at an incubation temperature of *T*_1_ and *T*_2_ (°C), respectively.

### 2.4. Statistical Analysis

One-way ANOVAs were performed using IBM SPSS Statistics 22 (SPSS Inc., Chicago, IL, USA) to analyze differences in plant traits across land use types, as well as differences in environmental conditions, CH_4_ and CO_2_ emissions, and their *Q*_10_ values among soil depths and land use types. General linear regression analyses were performed using Origin 2025 (OriginLab Corporation, Northampton, MA, USA) to characterize latitudinal patterns of environmental conditions, plant traits, and CH_4_ and CO_2_ emissions and their *Q*_10_ values. General linear regression analyses were employed to characterize the relationships between CH_4_ and CO_2_ emissions and their *Q*_10_ values and plant traits. Multiple linear regression analyses were performed with the “stats” package (Version 4.1.3) (R Core Team 2024) to analyze the effects of land use and climate and their interaction on the CH_4_ and CO_2_ emissions and their *Q*_10_ values. Linear mixed-effect models were performed with the “lme4” package (Version 1.1-30) [38] to evaluate the relative importance of environmental conditions for plant traits. The fixed-effect terms were environmental conditions, the random-effect factors were tidal inundation conditions, and the variance inflation factor (VIF) ≥  5 was excluded to avoid multicollinearity before linear mixed-effect models. Linear mixed-effect models were also performed to evaluate the relative importance of plant traits for CH_4_ and CO_2_ emissions and their *Q*_10_ values. The fixed-effect terms were plant traits, the random-effect factors were tidal inundation conditions, and the variance inflation factor (VIF) ≥  5 was excluded to avoid multicollinearity before linear mixed-effect models. To examine the direct and indirect effects of environmental conditions and plant traits on CH_4_ and CO_2_ emissions and their *Q*_10_ values, we performed a piecewise structural equation model using the “piecewiseSEM” package (Version 2.1.2) [39]. Pearson correlation analysis and mantel tests were performed with the “corrplot” package (Version 0.92) [40], “vegan” package (Version 2.5-6) [41], and “psych” package (Version 2.2.3) [42], respectively, to explore relationships between plant traits and analyze the relationships between plant traits and CH_4_ and CO_2_ emissions and their *Q*_10_ values in different land uses. All these statistical analyses were performed using R version 3.4.8.

## 3. Results

### 3.1. Latitudinal Patterns and Distributions of Carbon Emission

**Figure 2:**
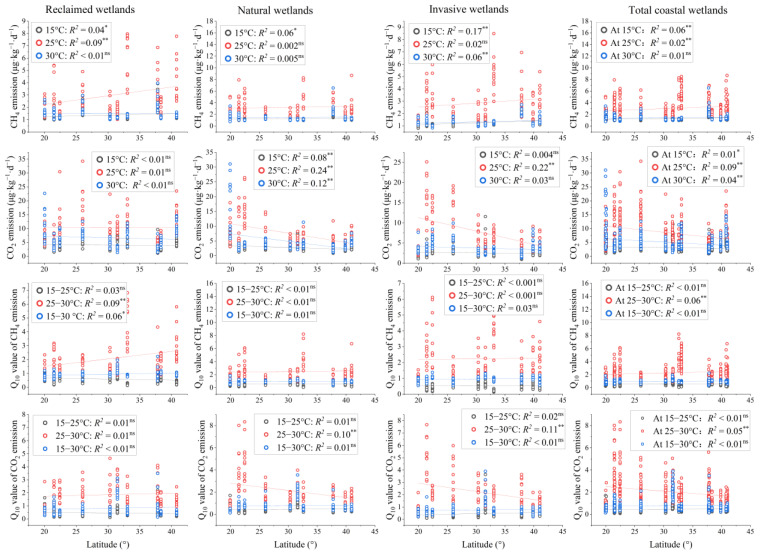
Relationships between CH_4_ and CO_2_ emissions and their *Q*_10_ values and latitude in different coastal land uses. * *p* < 0.05; ** *p* < 0.01; ns, *p* > 0.05.

**Figure 3:**
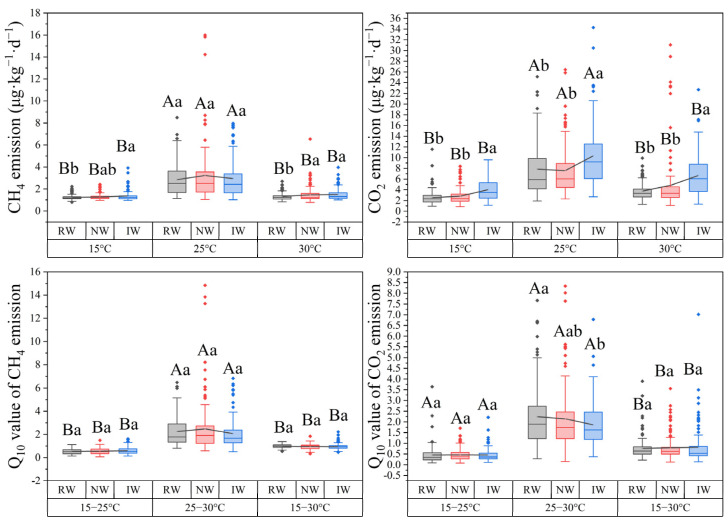
Distribution of CH_4_ and CO_2_ emissions and their *Q*_10_ values among different land uses (*p* < 0.05). Different lowercase letters indicate a significant difference in same incubation temperatures among different land uses (*p* < 0.05). Different uppercase letters indicate a significant difference in same land uses among different incubation temperatures (*p* < 0.05). RW, reclaimed wetlands. NW, natural wetlands. IW, invasive wetlands.

Land use, climate, and their interactions significantly affected soil carbon emissions, particularly CO_2_ emission (Table 2). However, the magnitudes and directions of these changes depended on incubation temperatures (Figure 2and Figure S2). Overall, latitudinal patterns of CH_4_ and CO_2_ emissions were stronger than those of their *Q*_10_ values (Figure 2). Significant patterns of CH_4_ emission were mainly shown at 15 °C and in IW (*p* < 0.05), while significant patterns of CO_2_ emission were mainly shown at 25 °C and in NW (*p* < 0.05) (Figure 3). Moreover, significant patterns of *Q*_10_ values were mainly displayed at 25–30 °C, and patterns of *Q*_10_ value of CH_4_ emission (*Q*_10_-CH_4_) were mainly exhibited in RW (*p* < 0.05), while patterns of *Q*_10_ value of CO_2_ emission (*Q*_10_-CO_2_) were mainly exhibited in NW and IW (*p* < 0.05).

Across all coastal herbaceous wetlands studied, CH_4_ and CO_2_ emissions were significantly higher at 25 °C, and their *Q*_10_ values were significantly higher at 25–30 °C (*p* < 0.05) (Figure 3). On average, CH_4_ and CO_2_ emissions were the largest in invasive wetlands, followed by natural wetlands, and the lowest in reclaimed wetlands, especially at 15 °C and 30 °C. Moreover, *Q*_10_-CH_4_ and *Q*_10_-CO_2_ were similar among these three land uses (*p* > 0.05).

### 3.2. The Response–Effect Trait Framework to Carbon Emission

**Figure 4:**
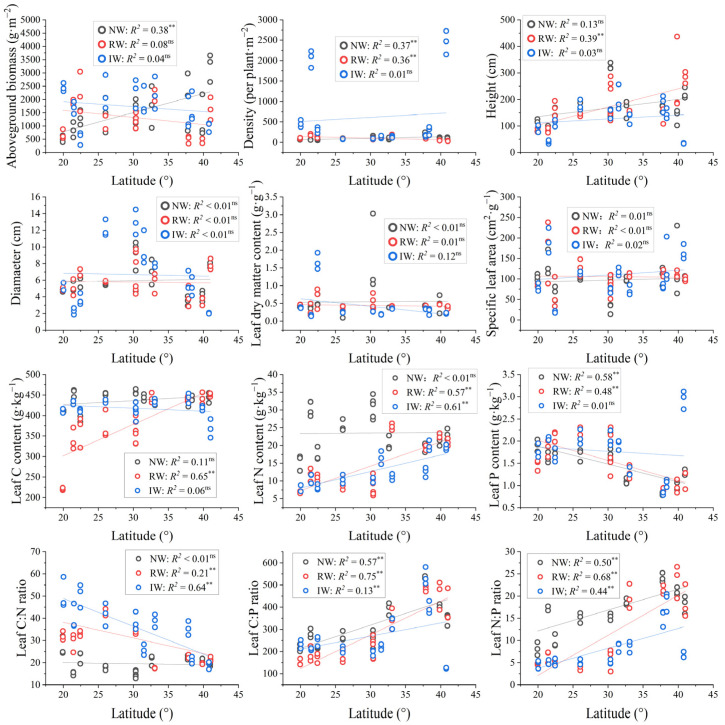
Relationships between plant traits and latitude in different coastal land uses. ** *p* < 0.01; ns, *p* > 0.05.

The latitudinal patterns of diameter, SLA, and LDMC were not significant (*p* > 0.05), but significant positive patterns of leaf C:P and N:P ratios were observed in each land use (*p* < 0.05) (Figure 4). On average, there were no significant differences in SLA, LDMC, or leaf P content and C:P ratio among the different land uses (*p* > 0.05) (Table 3). Compared with NW, IW had significantly higher AGB, density, diameter, and leaf C:N ratio, but significantly lower height, leaf N content, and N:P ratio (*p* < 0.05); in addition, RW had significantly lower AGB, leaf C and N contents, and N:P ratio, but significantly higher leaf C:N ratio (*p* < 0.05). Moreover, these changes were mainly driven by soil moisture and P content (Table S3).

**Figure 5:**
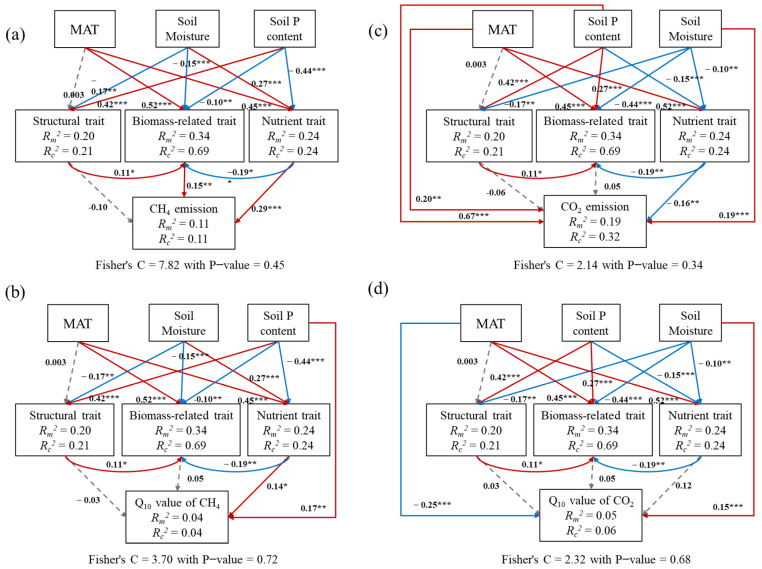
Structural equation modeling (SEM) results of influential pathways on CH_4_ and CO_2_ emissions and their Q_10_ values. MAT represents PC1 from PCA conducted with mean annual temperature and precipitation. Soil moisture and soil P content represents PC1 and PC2 from PCA, respectively, which was conducted with soil moisture, pH, salinity, bulk density, sulfate concentration, dissolved carbon content, and N and P contents. Plant-biomass-related trait represents PC1 from PCA conducted with diameter and height. Plant structural trait represents PC1 from PCA conducted with SLA and LDMC. Plant nutrient trait represents PC1 from PCA conducted with leaf C content and N:P ratio. Goodness-of-fit statistics for the model are shown below the models. *R_m_*^2^ represents marginal *R*^2^. *R_c_*^2^ represents conditional *R*^2^. Gray arrows with dashed lines represent non-significant pathways. Blue or red solid lines represent significant negative or positive pathways. * *p* < 0.05; ** *p* < 0.01; *** *p* < 0.001. SEM for each plant trait and soil CH_4_ and CO_2_ emissions and their *Q*_10_ values are shown in Figures S6–S9. (**a**) reclaimed wetlands; (**b**) natural wetlands; (**c**) invasive wetlands; (**d**) All the coastal wetlands.

Structural equation models showed that some plant traits affected CH_4_ and CO_2_ emissions and their *Q*_10_ values more strongly than other plant traits (Figure 5). Specifically, CH_4_ emission was mainly affected by leaf P content, C:P ratio, and N:P ratio (*p* < 0.05) (Figure S3); CO_2_ emission was mainly affected by leaf C, N, and P contents and ratios (*p* < 0.05) (Figure S4); and *Q*_10_-CH_4_ and *Q*_10_-CO_2_ were mainly affected by leaf N content and C:N and N:P ratios (*p* < 0.05) (Figures S5 and S6). In these SEMs, environmental conditions (e.g., MAT, soil P content, and moisture) also significantly influenced soil carbon emissions.

### 3.3. The Relationships Between Plant Traits and Carbon Emissions

**Figure 6:**
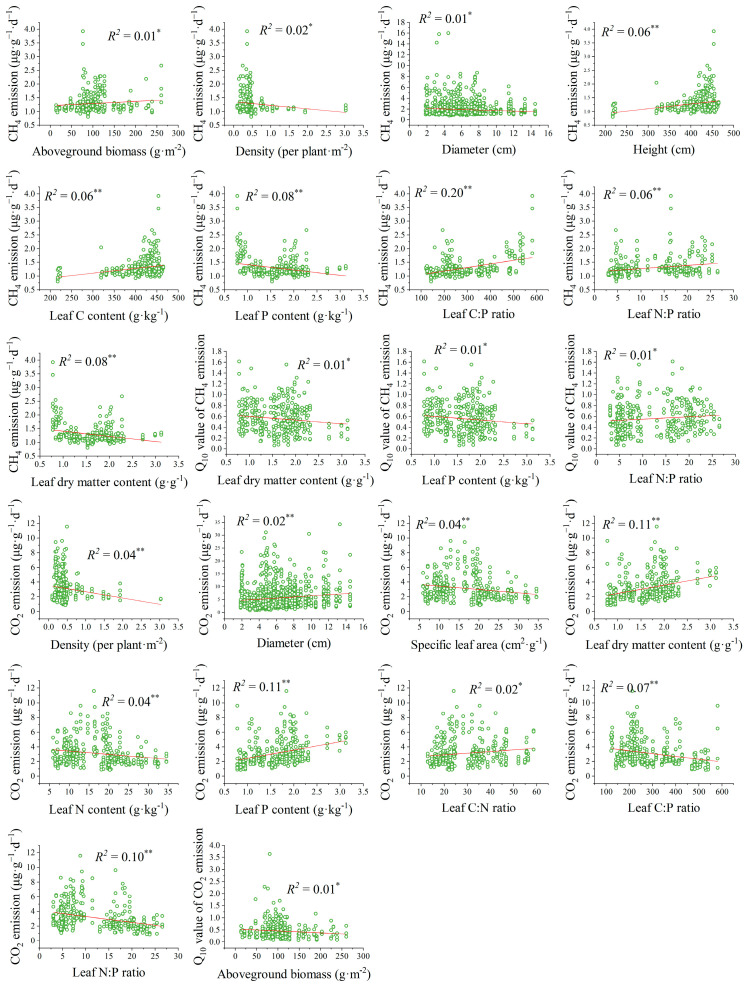
Significant relationships between plant traits and soil carbon emissions. * *p* < 0.05; ** *p* < 0.01; ns, *p* > 0.05.

General linear regressions showed that, except SLA, leaf N content, and C:N ratio, other plant traits were significantly correlated with soil CH_4_ emission (*p* < 0.05) (Figure 6 and Figure S7), and *Q*_10_-CH_4_ was significantly correlated with LDMC, leaf P content, and N:P ratio (*p* < 0.05) (Figure 6 and Figure S8). Aside from AGB and height, other plant traits were significantly correlated with CO_2_ emission (*p* < 0.05) (Figure 6 and Figure S9), and *Q*_10_-CO_2_ was only significantly correlated with AGB (*p* < 0.05) (Figure 6 and Figure S10).

**Figure 7:**
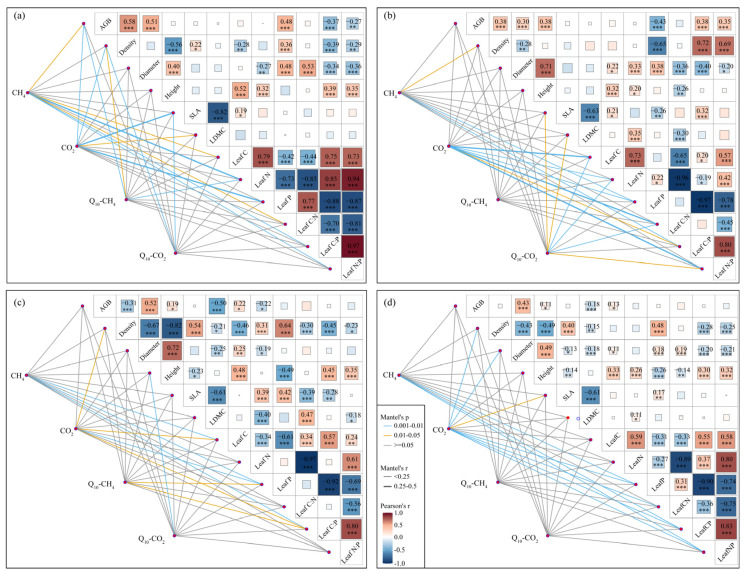
Relationships between plant traits and a mantel test between plant traits and soil CH_4_ and CO_2_ emissions and their *Q*_10_ values among different land uses. (**a**) Reclaimed wetlands. (**b**) Natural wetlands. (**c**) Invasive wetlands. (**d**) All the coastal wetlands. * *p* < 0.05; ** *p* < 0.01; *** *p* < 0.01.

Mantel tests showed that CH_4_ and CO_2_ emissions strongly correlated with leaf C, N, and P contents and ratios, while their *Q*_10_ values mainly related to biomass-related and structural traits (Figure 7). Pearson correlation analysis showed that the relationships between these nutrient traits were stronger in reclaimed wetlands than in both invasive and natural wetlands (Figure 7).

## 4. Discussion

### 4.1. The Response–Effect Trait Framework to Carbon Emissions

As expected, applying the “response–effect” trait framework to carbon emissions has improved predictability and enhanced generalizability in wetland ecosystems [20,21,43]. First, we exhibited that the response traits to environmental conditions and the effect traits of carbon emissions can be different or the same [20,25,43]. Across all of the coastal herbaceous wetlands studied, both biomass-related traits and structural traits are greatly affected by environmental conditions; however, biomass-related traits can only predict CH_4_ emission and structural traits can predict neither CH_4_ emission nor CO_2_ emission, which might limit our ability to understand carbon emissions. Interestingly, nutrient traits responded to environmental conditions and predicted CH_4_ and CO_2_ emissions and their *Q*_10_ values (Figure 5). Moreover, plant nutrient traits were strongly correlated with CH_4_ and CO_2_ emissions and their *Q*_10_ values (Figure 6). One potential explanation is that plant-derived nutrient quality significantly influenced microbial carbon metabolism [44] and then regulated the gaseous end products of organic matter decomposition [4].

Furthermore, we found that the effect traits regulating CH_4_ and CO_2_ emissions and their *Q*_10_ values can also be the same or different [20,43]. Specifically, CH_4_ and CO_2_ emissions and their *Q*_10_ values were all significantly predicted by AGB and leaf N:P ratio, which in turn were altered by environmental conditions (Figures S3–S6). Meanwhile, effect traits of different carbon emissions can also be different. For example, leaf N content and C:P ratio could significantly predict CH_4_ emission but not CO_2_ emission (Figures S3 and S5). For another instance, leaf C and P contents could significantly predict CH_4_ emission but not *Q*_10_-CH_4_ and *Q*_10_-CO_2_ (Figures S3, S4, and S6). These results indicated that both the quantity and quality of plant litter strongly affected the composition and activity of microbial communities and further regulated the CH_4_ and CO_2_ emissions in the soil and atmosphere [21,44,45]. Notably, there were situations where a trait influenced carbon emissions through another trait that predicted the carbon sink function [25]. According to the “dilution effect” theory [46], large individual biomass could decrease leaf nutrient contents and then affect leaf nutrient ratios. Our study showed that both biomass-related traits and structural traits were strongly correlated with nutrient traits (Figure 7), and nutrient traits were significantly predicted carbon emissions (Figure 5). These findings suggested that biomass-related and structural traits could regulate CH_4_ and CO_2_ emissions by mediating nutrient traits. These results also highlighted that capturing multiple traits proved to be more powerful in explaining CH_4_ and CO_2_ emissions in wetland ecosystems.

### 4.2. Effects of Environmental Conditions

In addition to plant traits, environmental conditions also strongly affected carbon emissions, and temperature showed strong effect (Figure 5 and Figures S3–S6). Across all of the coastal wetland studies, both CH_4_ and CO_2_ emissions were higher at 25 °C than at 15 °C and 30 °C, and their *Q*_10_ values were significantly higher at 25–30 °C than at 15–25 °C and 15–30 °C (Figure 3). These results indicated that the optimum temperature for methanogens was 25 °C [33], and most of the overall temperature sensitivity occurred at progressively higher temperatures [7]. Moreover, latitudinal patterns of CH_4_ and CO_2_ emissions were stronger at 25 °C and patterns of *Q*_10_-CH_4_ and *Q*_10_-CO_2_ were stronger at 25–30 °C (Figure 2). It is expected that both geochemical and biological processes occur at faster rates in the warmer and wetter regions than in the cooler and drier regions, resulting in rapid plant growth, intense soil weathering and erosion, and strong microbial activity [47,48]. Partly consistent with previous findings [10,49], significant patterns showed that CO_2_ emission and *Q*_10_-CO_2_ significantly increased, while CH_4_ emission and *Q*_10_-CH_4_ decreased with increasing latitude in coastal herbaceous wetlands. These findings were mainly attributed to the balance of CH_4_ production and oxidation in anaerobic soils [50]. A higher sulfate concentration combined with salinity could enhance the competitive effect on the competitive methanogenic substrate of sulfate-reducing bacteria relative to methanogens and then reduce the CH_4_ production [51]. On the other hand, the N-sufficient and high-sulfate soils could stimulate the process of anaerobic CH_4_ oxidation [52,53]. The high-sulfate and N-rich conditions in lower latitudes were not conducive to CH_4_ production, but favored CH_4_ oxidation to produce CO_2_ emission, and consequently formed a positive latitudinal pattern of CH_4_ emission but negative latitudinal pattern of CO_2_ emission (Figure 2).

### 4.3. Effects of Land Uses on Carbon Emission

The relationships between plant traits and carbon emissions varied greatly with costal land uses (Figure 7). Moreover, plant traits were mainly affected by soil moisture and nutrient conditions in coastal wetlands (Table S3). Compared to those in natural wetlands, soil moisture and N and P contents in reclaimed wetlands were significantly lower (Table S2), which was likely attributable to the disappearance of additional material exchanges from seawater caused by seawalls [35] and might affect plant nutrient absorption and nutrient use efficiency [29,30,37,54]. As an indicator of nutrient limitation [55,56], the leaf N:P ratio was significantly and negatively correlated with AGB (Figure 7a), indicating that plant growth of reclaimed wetlands was limited by N nutrient. Thus, reclaimed wetlands supported a *P. australis* community with significantly lower AGB, leaf C and N contents, and N:P ratios but significantly higher leaf C:N ratio relative to those of *P. australis* in natural wetlands (Table 3). It is generally reported that CO_2_ and especially CH_4_ emissions in anaerobic conditions were carbon limited [20,36]; therefore, the significantly lower C content and AGB of plants eventually decreased both CH_4_ and CO_2_ emissions in reclaimed wetlands relative to those of natural wetlands (Figure 3).

In addition, variations in CH_4_ and CO_2_ emissions in invasive wetlands were mainly driven by leaf C and P contents and C:P ratios (Figure 7c), but these traits were similar to those in natural wetlands (Table 3). An invasive *S. alterniflora* community had lower leaf N content and N:P ratio but higher C:N ratio relative to that of native *P. australis* in natural wetlands (Figure 4). Significantly higher salinity and pH in low-tide areas might restrict microbial activity and then decrease soil N and P availability [57] and might affect nutrient absorption of *S. alterniflora* [29]. However, the additional nutrients from seawater through irregular tidal erosion might make *S. alterniflora* not lack N or P nutrients [35,58]. Moreover, the leaf N:P ratio was not correlated with AGB in invasive wetlands (Figure 7c), suggesting that the growth of *S. alterniflora* was not limited by N or P nutrients [55,56]. Although AGB was not associated with either CH_4_ or CO_2_ emissions in invasive wetlands (Figure 7c), their values were the largest (Table 3), and the CH_4_ and especially CO_2_ emissions were also the highest compared to those of the other coastal herbaceous wetlands (Figure 3).

### 4.4. Limitations and Future Recommendations

Previous studies showed strong relationships between plant traits and CH_4_ and CO_2_ emissions from wetland soils in both field and laboratory conditions [20,22,43]. In this study, however, we only provided indirect evidence that CH_4_ and CO_2_ emissions obtained through laboratory incubation experiments were closely related to the plant traits in the tested wetlands at a geographical scale. Moreover, soil carbon emissions are primarily driven by microbial processes [50], and our other research results showed that the diversity of methanogenic archaea and sulfate-reducing bacteria was closely related to plant traits, as well as CH_4_ and CO_2_ emissions from coastal wetland soils. Hence, future studies could well establish a direct linkage between the carbon emissions from soil microbial metabolism and plant traits, and, therefore, help to accurately and mechanistically understand the implications of plant traits for soil carbon emissions in coastal herbaceous wetlands.

## 5. Conclusions

Overall, the “response–effect” trait framework provided insights into mechanisms of anaerobic carbon emissions from coastal soils, with plant nutrient traits serving as strong predictors for both CH_4_ and CO_2_ emissions. These traits tended to exhibit significant correlations with biomass-related and structural traits, with these relationships being further influenced by environmental conditions. Notably, it should be highlighted that the relationships between response and effect traits observed in coastal ecosystems depend on a very limited number of plant species in our study. Specifically, with the process of vegetation succession, especially in reclaimed wetlands, vegetation community and specific trait composition will be changed, and, therefore, have the potential to influence the relationships between environment and soil carbon decomposition considerably, and consequently have a profound impact on the carbon function of the whole coastal ecosystem.

## Figures and Tables

**Table 1:** Plant traits (a), environmental conditions (b), and carbon emissions (c).

	Distribution	Unit
(a) Plant traits		
Specific leaf area (SLA)	Structural trait	cm^2^·g^−1^
Leaf dry matter content (LDMC)	Structural trait	g·g^−1^
Density	Biomass-related trait	per plant·m^−2^
Diameter	Biomass-related trait	cm
Height	Biomass-related trait	cm
Aboveground biomass (AGB)	Biomass-related trait	g·m^−2^
Leaf C content	Nutrient trait	g·kg^−1^
Leaf N content	Nutrient trait	g·kg^−1^
Leaf P content	Nutrient trait	g·kg^−1^
Leaf C:N ratio	Nutrient trait	——
Leaf C:P ratio	Nutrient trait	——
Leaf N:P ratio	Nutrient trait	——
(b) Environmental conditions		
Mean annual temperature (MAT)	Climate	°C
Mean annual precipitation (MATP)	Climate	mm
Moisture	Soil properties	%
pH	Soil properties	——
Salinity	Soil properties	g·kg^−1^
Sulfate concentration	Soil properties	mg·g^−1^
Dissolved carbon content (DOC)	Soil properties	mg·kg^−1^
Soil N content	Soil properties	g·kg^−1^
Soil P content	Soil properties	g·kg^−1^
(c) Soil carbon emissions		
CH_4_ emission	Carbon emission	μg·g^−1^·d^−1^
CO_2_ emission	Carbon emission	μg·g^−1^·d^−1^
*Q*_10_ value of CH_4_ emission (*Q*_10_-CH_4_)	Temperature sensitivity	——
*Q*_10_ value of CO_2_ emission (*Q*_10_-CO_2_)	Temperature sensitivity	——

**Table 2:** Interactive effects of mean annual temperature (MAT, left column) and precipitation (MAP, right column) with land uses on CH_4_ and CO_2_ emissions and their *Q*_10_ values. Direction of effects are marked in bold (negative effect = blue shaded, positive effect = light pink shaded). Significant effects are represented in bold. RW, reclaimed wetlands. NW, natural wetlands. IW, invasive wetlands.

	CH_4_	CO_2_	*Q*_10_-CH_4_	*Q*_10_-CO_2_		CH_4_	CO_2_	*Q*_10_-CH_4_	*Q*_10_-CO_2_
Intercept (NW)	**2.076**	**−0.358**	**1.391**	**0.571**	Intercept (NW)	**2.236**	**1.417**	**1.232**	**0.675**
RW	0.183	1.585	−0.067	0.104	RW	−0.088	0.675	−0.065	0.230
IW	0.489	7.122	0.307	**0.572**	IW	0.256	**4.777**	0.223	**0.462**
MAT	−0.005	0.315	−0.004	**0.033**	MAP	<0.001	**0.003**	<0.001	**<0.001**
RW × MAT	−0.022	−0.110	−0.001	−0.004	RW × MAP	<0.001	−0.001	<0.001	<0.001
IW × MAT	−0.032	−0.300	**−0.026**	**−0.038**	IW × MAP	<0.001	**−0.002**	**<0.001**	**<0.001**
*R*^2^*_adj_*-squared	0.030	0.259	0.022	0.038	*R*^2^*_adj_*-squared	0.055	0.273	0.014	0.042
F-statistic	3.227	26.170	2.584	3.811	F-statistic	5.191	27.890	2.027	4.122
*p*-value	0.007	<0.001	0.026	0.002	*p*-value	0.001	<0.001	0.074	0.001

**Table 3:** Distribution of plant traits among different land uses (*p* < 0.05). RW, reclaimed wetlands. NW, natural wetlands. IW, invasive wetlands. Different lowercase letters indicate a significant difference among different land uses (*p* < 0.05).

	Natural Wetlands	Reclaimed Wetlands	Invasive Wetlands
Aboveground biomass (g·m^−2^)	1518.95 ± 857.52 ^ab^	1318.38 ± 686.23 ^b^	1730.69 ± 702.62 ^a^
Density (per plant·m^−2^)	102.17 ± 43.40 ^b^	101.90 ± 49.50 ^b^	614.13 ± 841.12 ^a^
Diameter (cm)	5.95 ± 1.92 ^a^	5.77 ± 1.76 ^a^	6.68 ± 3.78 ^a^
Height (cm)	168.40 ± 66.15 ^a^	173.46 ± 80.84 ^a^	127.20 ± 58.21 ^b^
Specific leaf area (cm^2^·g^−1^)	97.14 ± 38.98 ^a^	105.68 ± 37.95 ^a^	110.14 ± 58.60 ^a^
Leaf dry matter content (g·g^−1^)	0.55 ± 0.51 ^a^	0.45 ± 0.15 ^a^	0.42 ± 0.43 ^a^
Leaf C content (g·kg^−1^)	436.82 ± 20.98 ^a^	379.66 ± 69.51 ^b^	417.72 ± 22.37 ^a^
Leaf N content (g·kg^−1^)	23.54 ± 5.66 ^a^	14.36 ± 6.73 ^b^	12.80 ± 4.23 ^b^
Leaf P content (g·kg^−1^)	1.48 ± 0.39 ^b^	1.55 ± 0.48 ^ab^	1.77 ± 0.59 ^a^
Leaf C:N ratio	19.51 ± 4.19 ^b^	30.97 ± 11.46 ^a^	36.15 ± 11.13 ^a^
Leaf C:P ratio	321.47 ± 101.59 ^a^	278.84 ± 128.30 ^a^	271.01 ± 115.87 ^a^
Leaf N:P ratio	16.94 ± 4.88 ^a^	11.38 ± 8.05 ^b^	8.32 ± 4.79 ^b^

## Data Availability

The original contributions presented in this study are included in the article and Supplementary Materials. Further inquiries can be directed to the corresponding authors.

## Supplementary Materials

The following supporting information can be downloaded at: https://www.mdpi.com/article/10.3390/plants14182852/s1. Table S1: Distribution of the sampling sites in Chinese coastal wetlands. Table S2: Soil properties of different soil depths of different land uses. Table S3: Summary of linear mixed-effects models for the effects of environmental conditions on the plant traits across three land uses. Figure S1: Relationships between soil properties and latitude. Figure S2: Distribution of CH_4_ and CO_2_ emissions and their Q_10_ values among different soil depths, incubation temperatures, and land uses. Figure S3: Structure equation modeling (SEM) results of influential pathways on CH_4_ emission. Figure S4: Structure equation modeling (SEM) results of influential pathways on CO_2_ emission. Figure S5: Structure equation modeling (SEM) results of influential pathways on the Q_10_ value of CH_4_ emission. Figure S6: Structure equation modeling (SEM) results of influential pathways on the Q_10_ value of CO_2_ emission. Figure S7: Linear relationships between plants and CH_4_ emission. Figure S8: Linear relationships between plants and Q_10_ value of CH_4_ emission. Figure S9: Linear relationships between plants and CO_2_ emission. Figure S10: Linear relationships between plants and Q_10_ value of CO_2_ emission.

## References

[1] Wang F.M., Liu J.H., Qin G.M., Zhang J.F., Zhou J.G., Wu J.T., Zhang L.L., Thapa P., Sanders C.J., Santos I.R. (2023). Coastal blue carbon in China as a nature-based solution towards carbon neutrality. Innovation.

[2] Mcleod E., Chmura G.L., Bouillon S., Salm R., Björk M., Duarte C.M., Lovelock C.E., Schlesinger W.H., Silliman B.R. (2011). A blueprint for blue carbon: Toward an improved understanding of the role of vegetated coastal habitats in sequestering CO_2_. Front. Ecol. Environ..

[3] Hansen J.E., Lacis A.A. (1990). Sun and dust versus greenhouse gases: An assessment of their relative roles in global climate change. Nature.

[4] Yvon-Durocher G., Montoya J.M., Woodward G., Jones J.I., Trimmer M. (2011). Warming increases the proportion of primary production emitted as methane from freshwater mesocosms. Glob. Change Biol..

[5] Yang H.L., Tang J.W., Zhang C.S., Dai Y.H., Zhou C., Xu P., Perry D.C., Chen X.C. (2020). Enhanced carbon uptake and reduced methane emissions in a newly restored wetland. J. Geophys. Res. Biogeosci..

[6] Pachauri R.K., Meyer L.A., IPCC, Core Writing Team (2014). Climate change 2014: Synthesis report. Contribution of Working Groups I, II and III to the Fifth Assessment Report of the Intergovernmental Panel on Climate Change.

[7] Inglett K.S., Inglett P.W., Reddy K.R., Osborne T.Z. (2012). Temperature sensitivity of greenhouse gas production in wetland soils of different vegetation. Biogeochemistry.

[8] Chen H.Y., Xu X., Fang C.M., Li B., Nie M. (2021). Differences in the temperature dependence of wetland CO_2_ and CH_4_ emissions vary with water table depth. Nat. Clim. Change.

[9] Nie M. (2020). Hydrospheric methane emission and its microbiological mechanisms under climate warming. Acta Microbiol. Sin..

[10] Li X.F., Sardans J., Hou L.J., Liu M., Xu C.B., Peñuelas J. (2020). Climatic temperature controls the geographical patterns of coastal marshes greenhouse gases emissions over China. J. Hydrol..

[11] Chen H.Y., Zhu T., Li B., Fang C.M., Nie M. (2020). The thermal response of soil microbial methanogenesis decreases in magnitude with changing temperature. Nat. Commun..

[12] Zhang H.J., Yao X.D., Zeng W.J., Fang Y., Wang W. (2020). Depth dependence of temperature sensitivity of soil carbon dioxide, nitrous oxide and methane emissions. Soi. Biol. Biochem..

[13] Hu M.J., Sardans J., Yang X.Y., Peñuelas J., Tong C. (2020). Patterns and environmental drivers of greenhouse gas fluxes in the coastal wetlands of China: A systematic review and synthesis. Environ. Res..

[14] Girkin N.T., Dhandapani S., Evers S., Ostle N., Turner B.L., Sjögerste S. (2020). Interactions between labile carbon, temperature and land use regulate carbon dioxide and methane production in tropical peat. Biogeochemistry.

[15] Kooch Y., Ghorbanzadeh N., Francaviglia R. (2022). Soil carbon stocks can be negatively affected by land use and climate change in natural ecosystems of semi-arid environment of Iran. Geoderma Reg..

[16] Davidson N.C. (2014). How much wetland has the world lost? Long-term and recent trends in global wetland area. Mar. Freshw. Res..

[17] Ma Z.J., Melville D.S., Liu J.G., Chen Y., Yang H.Y., Ren W.W., Zhang Z.W., Piersma T., Li B. (2014). Ecosystems management rethinking China’s new great wall. Science.

[18] Hu Y.K., Tian B., Yuan L., Li X.Z., Huang Y., Shi R.H., Jiang X.Y., Wang L.H., Sun C. (2021). Mapping coastal salt marshes in China using time series of Sentinel-1 SAR. ISPRS J. Photogramm. Remote Sens..

[19] Yuan J.J., Liu D.Y., Ji Y., Xiang J., Lin Y.X., Wu M., Ding W.X. (2019). *Spartina alterniflora* invasion drastically increases methane production potential by shifting methanogenesis from hydrogenotrophic to methylotrophic pathway in a coastal marsh. J. Ecol..

[20] Sutton-Grier A.E., Megonigal J.P. (2011). Plant species traits regulate methane production in freshwater wetland soils. Soil Biol. Biochem..

[21] Happonen K., Virkkala A.M., Kemppinen J., Niittynen P., Luoto M. (2021). Relationships between above-ground plant traits and carbon cycling in tundra plant communities. J. Ecol..

[22] Goud E.M., Moore T.R., Roulet N.T. (2017). Predicting peatland carbon fluxes from non-destructive plant traits. Funct. Ecol..

[23] Díaz S., Kattge J., Cornelissen J., Wright I., Lavorel S., Dray S., Reu B., Kleyer M., Writh C., Prentice I. (2016). The global spectrum of plant form and function. Nature.

[24] Díaz S., Cabido M. (2001). Vive la difference: Plant functional diversity matters to ecosystem processes. Trends Ecol. Evol..

[25] Minden V., Kleyer M. (2015). Ecosystem multifunctionality of coastal marshes is determined by key plant traits. J. Veg. Sci..

[26] Zibel C.R., Bassett T., German E., Brudvig L.A. (2017). Plant functional traits and environmental conditions shape community assembly and ecosystem functioning during restoration. J. Appl. Ecol..

[27] Wright I.J., Reich P.B., Westoby M., Ackerly D.D., Baruch Z., Bongers F., Cavender-Bares J., Chapin T., Comelissen J.H.C., Diemer M. (2004). The worldwide leaf economics spectrum. Nature.

[28] Girard A., Schweiger A.K., Carteron A., Kalacska M., Laliberté E. (2020). Foliar spectra and traits of bog plants across nitrogen deposition gradients. Remote Sens..

[29] Xiong J., Shao X.X., Yuan H.J., Liu E.J., Xu H.D., Wu M. (2024). Effect of human reclamation and *Spartina alterniflora* invasion on C-N-P stoichiometry in plant organs across coastal wetlands over China. Plant Soil..

[30] Xiong J., Shao X.X., Li N., Yuan H.J., Liu E.J., Wu M. (2024). Effects of land-use on soil C, N, and P stocks and stoichiometry in coastal wetlands dependent on soil depth and latitude. Catena.

[31] Ren G.Q., Cui M.M., Yu H.C., Fan X., Zhu Z.Q., Zhang H.Y., Dai Z.C., Sun J.F., Yang B., Du D.L. (2024). Global environmental change shifts ecological stoichiometry coupling between plant and soil in early-stage invasions. J. Soil. Sci. Plant Nutr..

[32] Jackson M.L. (1958). Soil Chemical Analysis.

[33] Wang W., Zeng C., Sardans J., Wang C., Tong C., Peñuelas J. (2018). Soil methane production, anaerobic and aerobic oxidation in porewater of wetland soils of the Minjiang River Estuarine, China. Wetlands.

[34] Reinhardt C.H., Cole C.A., Stover L.R. (2000). A method for coring inland, freshwater wetland soils. Wetlands.

[35] Xu X., Liu H., Liu Y.Z., Zhou C.H., Pan L.H., Fang C.M., Nie M., Li B. (2020). Human eutrophication drives biogeographic saltmarsh productivity patterns in China. Ecol. Appl..

[36] Megonigal J.P., Hines M.E., Visscher P.T., Schlesinger W.H. (2004). Anaerobic metabolism: Linkages to trace gases and aerobic processes. Biogeochemistry.

[37] Xiong J., Shao X., Yuan H., Liu E., Xu H., Wu M. (2022). Carbon, nitrogen, and phosphorus stoichiometry and plant growth strategy as related to land-use in Hangzhou Bay coastal wetland, China. Front. Plant Sci..

[38] Bates D., Mächler M., Bolker B., Walker S. (2015). Fitting linear mixed-effects models using lme4. J. Stat. Softw..

[39] Lefcheck J.S. (2016). piecewiseSEM: Piecewise structural equation modeling in R for ecology, evolution, and systematics. Methods Ecol. Evol..

[40] Wei T.Y., Simko V. (2021). R Package ‘Corrplot’: Visualization of a Correlation Matrix.

[41] Oksanen J., Simpson G., Blanchet F., Kindt R., Legendre P., Minchin P., O’Hara R., Solymos P., Stevens M., Szoecs E. (2022). Vegan: Community Ecology Package.

[42] Revelle W. (2022). Psych: Procedures for Psychological, Psychometric, and Personality Research.

[43] Goud E.M., Touchette S., Strachan I.B., Strack M. (2022). Graminoids vary in functional traits, carbon dioxide and methane fluxes in a restored peatland: Implications for modelling carbon storage. J. Ecol..

[44] Xu Z.W., Yu G.R., Wang Q.F., Zhang X.Y., Wang R.L., Zhao N., He N.P., Liu Z.P. (2019). Plant functional traits determine latitudinal variations in soil microbial function: Evidence from forests in China. Biogeosciences.

[45] Lau J.A. (2011). Aboveground–Belowground Linkages: Biotic Interactions, Ecosystem processes, and Global Change. Q. Rev. Biol..

[46] Reich P.B., Ellsworth D.S., Uhl C. (1995). Leaf carbon and nutrient assimilation and conservation in species of differing successional status in an oligotrophic Amazonian forest. Funct. Ecol..

[47] Li X.Q., Xia J.B., Zhao X.M., Chen Y.P. (2019). Effects of planting *Tamarix* chinensis on shallow soil water and salt content under different groundwater depths in the Yellow River Delta. Geoderma.

[48] Tian H., Chen G., Zhang C., Melillo J.M., Hall C.A. (2010). Pattern and variation of C: N:P ratios in China’s soils: A synthesis of observational data. Biogeochemistry.

[49] Zhu D., Wu N., Bhattarai N., Oil K.P., Chen H., Rawat G.S., Rashid I., Dhakal M., Joshi S., Tian J.Q. (2021). Methane emissions respond to soil temperature in convergent patterns but divergent sensitivities across wetlands along altitude. Glob. Change Biol..

[50] Dean J.F., Middelburg J.J., Röckmann T., Aerts R., Blauw L.G., Egger M., Jetten M.S.M., de Jong A.E.E., Msisel O.H., Rasigraf O. (2018). Methane feedbacks to the global climate system in a warmer world. Rev. Geophys..

[51] Mishra S.R., Pattnaik P., Sethunathan N., Adhya T.K. (2003). Anion-mediated salinity affecting methane production in a flooded alluvial soil. Geomicrobiol. J..

[52] Segarra K.E.A., Schubotz F., Samarkin V., Yoshinaga M.Y., Hinrichs K.U., Joye S.B. (2015). High rates of anaerobic methane oxidation in freshwater wetlands reduce potential atmospheric methane emissions. Nat. Comm..

[53] Shen L.D., Hu B.L., Liu S., Chai X.P., He Z.F., Ren H.X., Liu Y., Geng S., Wang W., Tang J.L. (2016). Anaerobic methane oxidation coupled to nitrite reduction can be a potential methane sink in coastal environments. Appl. Genet. Mol. Biotechnol..

[54] Xiong J., Sheng X.C., Wang M., Wu M., Shao X.X. (2022). Comparative study of methane emission in the reclamation-restored wetlands and natural marshes in the Hangzhou Bay coastal wetland. Ecol. Eng..

[55] Yan Z.B. (2017). Effects of nitrogen and phosphorus addition on the transgenerational growth and stoichiometry of *Arabidopsis thaliana*. Ph.D. dissertation.

[56] Güsewell S. (2004). N:P ratios in terrestrial plants: Variation and functional significance. New Phytol..

[57] van Dijk G., Lamers L.P.M., Loeb R., Westendorp P.J., Kuiperij R., van Kleef H.H., Klinge M., Smolders A.J.P. (2019). Salinization lowers nutrient availability in formerly brackish freshwater wetlands; unexpected results from a long-term field experiment. Biogeochemistry.

[58] Dierberg F.E., DeBusk T.A., Larson N.R., Kharbanda M.D., Chan N., Gabriel M.C. (2011). Effects of sulfate amendments on mineralization and phosphorus release from South Florida (USA) wetland soils under anaerobic conditions. Soil. Biol. Biochem..

