# Supplementary material for: Geographical-Scale Evidence Reveals Plant Nutrient as an Effective Indicator for Coastal Carbon Emissions
Source: Plants (Basel). 2025 Sep 12;14(18):2852. doi: 10.3390/plants14182852 (PMC12473945; doi:10.3390/plants14182852)
Supplement: Supplementary file 1 [file plants-14-02852-s001.zip › plants-3805902-supplementary.pdf]

Geographical-Scale Evidence Reveals Plant Nutrient as an Effective Indicator for Coastal Carbon Emissions

Jing Xiong [1,2,3,4], Xuexin Shao [1,3,4,*], Haidong Xu [5] and Ming Wu [1,3,4,*]

[1] Research Institute of Subtropical Forestry, Chinese Academy of Forestry, Hangzhou 311400, China; xiongxiaojing2016@163.com
[2] School of Life Sciences, Jinggangshan University, Ji'an 343009, China
[3] Wetland Ecosystem Research Station of Hangzhou Bay, Research Institute of Subtropical Forestry,
Chinese Academy of Forestry, Hangzhou 311400, China
[4] State Key Laboratory of Wetland Conservation and Restoration, Beijing 100080, China
[5] Yellow River Delta Ecological Environment Research Center, Shandong University of Aeronautics,
Binzhou 256603, China; haidongxuu@163.com
* Correspondence: shaoxuexin@126.com (X.S.); hangzhoubay@126.com (M.W.)

Supplementary Material

Table S1: Distribution of the sampling sites in Chinese coastal wetlands.

Table S2: Soil properties of different soil depths of different land uses.

Table S3: Summary of linear mixed-effects models for the effects of environmental conditions on the plant traits across three land uses.

Figure S1: Relationships between soil properties and latitude.

Figure S2: Distribution of CH_4 and CO_2 emissions and their Q_{10} values among different soil depths, incubation temperatures, and land uses.

Figure S3: Structure equation modeling (SEM) results of influential pathways on CH_4 emission.

Figure S4: Structure equation modeling (SEM) results of influential pathways on CO_2 emission.

Figure S5: Structure equation modeling (SEM) results of influential pathways on the Q_{10} value of CH_4 emission.

Figure S6: Structure equation modeling (SEM) results of influential pathways on the Q_{10} value of CO_2 emission.

Figure S7: Linear relationships between plants and CH_4 emission.

Figure S8: Linear relationships between plants and Q_{10} value of CH_4 emission.

Figure S9: Linear relationships between plants and CO_2 emission.

Figure S10: Linear relationships between plants and Q_{10} value of CO_2 emission.

Table S1: Distribution of the sampling sites in Chinese coastal wetlands. IW, invasive wetlands. NW, natural wetlands. RW, reclaimed wetlands.

Site location	Land use	Latitude (°)	Longitude (°)	MAT (°C)	MAP (mm)	Plant species	Tide level
Haikou, Hainan	IW	E110.43	N20.05	23.5	1731.4	*S. alterniflora*	Low or middle tide
	NW	E110.63	N19.92	23.5	1731.4	*P. australis*, and mangrove	High tide
	RW	E110.56	N19.96	23.5	1731.4	*P. australis,*	Reclaimed areas
Zhanjiang, Guangdong	IW	E109.79	N21.51	23.3	1723	*S. alterniflora*	Low or middle tide
	NW	E109.92	N21.44	23.3	1723	*P. australis*, and mangrove	High tide
	RW	E109.91	N21.45	23.3	1723	*P. australis,*	Reclaimed areas
Zhuhai, Guangdong	IW	E113.63	N22.42	22.6	2082	S. alterniflora	Low or middle tide
	NW	E113.63	N22.43	22.6	2082	P. australis, and mangrove	High tide
	RW	E113.63	N22.42	22.6	2082	P. australis,	Reclaimed areas
Min River Estuary, Fujian	IW	E119.63	N26.03	19.9	1405.4	*S. alterniflora*	Low or middle tide
	NW	E119.61	N26.06	16.1	1304.5	*P. australis*	High tide
	RW	E119.64	N26.02	19.9	1405.4	*P. australis*	Reclaimed areas
Hangzhou Bay, Zhejiang	IW	E121.08	N30.32	16.1	1304.5	*S. alterniflora*	Low or middle tide
	NW	E121.08	N30.32	15.8	1128.9	*P. australis*	High tide
	RW	E121.14	N30.36	16.1	1304.5	*P. australis*	Reclaimed areas
Chongming, Shanghai	IW	E121.99	N31.50	15.8	1128.9	*S. alterniflora*	Low or middle tide
	NW	E121.89	N31.60	16.2	1118.3	*P. australis*	High tide
	RW	E121.92	N31.59	15.8	1128.9	*P. australis*	Reclaimed areas
Dafeng, Jiangsu	IW	E120.86	N33.07	14.4	1066.9	*S. alterniflora*	Low or middle tide
NanTong, Jiangsu	NW	E120.95	N32.61	15.8	1128.9	*P. australis*	High tide
Dafeng, Jiangsu	RW	E120.85	N33.06	14.4	1066.9	*P. australis*	Reclaimed areas
Yellow River Estuary, Shandong	IW	E119.07	N37.84	13.2	534.9	*S. alterniflora*	Low or middle tide
	NW	E118.82	N37.75	16.2	1118.3	*P. australis*	High tide
	RW	E120.85	N33.06	13.2	534.9	*P. australis*	Reclaimed areas
Cangzhou, Heibei	IW	E117.73	N38.35	11.4	536.7	*S. alterniflora*	Low or middle tide
Beidaihe, Hebei	NW	E119.52	N39.85	11.4	636.7	*P. australis*	High tide
	RW	E119.51	N39.85	11.4	636.7	*P. australis*	Reclaimed areas
Jinzhou, Liaoning	IW	E120.96	N10.80	9.8	577.4	*S. alterniflora*	Low or middle tide
Shuangtaizi Eastuary, Liaoning	NW	E121.82	N40.94	23.5	1731.4	*P. australis*	High tide
	RW	E121.87	N41.01	9.8	640.1	*P. australis*	Reclaimed areas

Table S2: Soil properties of different soil depths of different land uses (mean value ± standard deviation, n = 90). Different lower-case letters indicate a significant difference among soil depths in the same land use at *P* < 0.05. Different capital letters indicate a significant difference among land uses in the same soil depth at *P* < 0.05. IW, invasive wetlands. NW, natural wetlands. RW, reclaimed wetlands.

Land uses	Depth (cm)	Moisture (%)	pH	Salinity ($g·kg^{-1}$)	Bulk density ($g·cm^{-3}$)	Sulfate concentration ($mg·kg^{-1}$)	Dissolved C content ($mg·kg^{-1}$)	Soil N content ($g·kg^{-1}$)	Soil P content ($g·kg^{-1}$)
RW	0–10	32.59±14.19Ba	7.50±1.54Ba	5.24±4.19Ba	1.13±0.20Aa	0.69±0.61Ba	162.29±79.27Ba	0.78±0.35Ba	0.49±0.18Ba
	10–30	34.16±14.46Ba	7.71±1.46Ba	5.62±3.82Ba	1.12±0.18ABab	0.99±0.92Ba	153.26±90.75Ba	0.56±0.31Bb	0.42±0.17Aa
	30–60	37.90±16.77Ba	7.82±1.41Ba	5.72±3.95Ba	1.11±0.22Aa	0.99±0.85Ba	142.70±76.21Ba	0.55±0.29Bb	0.45±0.18Aa
	60–100	39.71±15.05Aa	7.79±1.29Ba	6.46±3.37Ba	1.08±0.27Aab	1.12±0.90Aa	157.99±95.84Aa	0.64±0.36Aab	0.46±0.14Aa
	Average	36.09±15.23B	7.70±1.41B	5.76±3.82C	1.11±0.22A	0.95±0.83B	152.89±84.41AB	0.63±0.34B	0.45±0.17B
NW	0–10	49.02±22.55ABa	7.89±0.79ABa	6.06±3.81Ba	1.09±0.25ABab	0.77±0.38Ba	155.58±87.45Ba	1.03±0.54Ab	0.59±0.22Aa
	10–30	48.22±21.37ABa	7.75±1.07Ba	6.65±4.44Ba	1.10±0.32ABab	1.29±1.28ABa	145.61±75.43Ba	0.78±0.40Ab	0.51±0.22Aa
	30–60	48.63±24.34Aa	7.86±0.88Ba	7.37±5.02Ba	1.11±0.25Aa	1.13±1.05Aa	126.71±83.52Ba	0.71±0.42Ab	0.52±0.21Aa
	60–100	48.25±24.14Aa	7.86±0.85Ba	7.35±4.83Ba	1.11±0.27Aab	1.24±1.03Aa	114.74±75.77Ba	0.64±0.44Ab	0.50±0.18Aa
	Average	48.53±22.84A	7.84±0.89B	6.86±4.52BC	1.11±0.27A	1.11±1.00B	128.59±74.51AB	0.79±0.48A	0.53±0.21A
IW	0–10	56.80±21.80Aa	8.03±0.51Aa	13.44±8.68Aa	1.00±0.20Bb	1.29±0.75Aa	194.65±99.57Aa	0.93±0.36Aa	0.54±0.15Aa
	10–30	52.53±19.72Aa	8.06±0.43Aa	12.65±7.24Ab	1.02±0.25Bb	1.58±0.84Aa	182.88±107.68Aa	0.74±0.33Ab	0.50±0.16Aa
	30–60	51.68±16.99Aa	8.09±0.56Aa	12.25±6.38Ab	1.01±0.22Ba	1.40±0.69Aa	168.68±83.27Aa	0.65±0.26Ab	0.48±0.18Aa
	60–100	46.50±16.46Aa	8.21±0.67Aa	12.82±7.38Ab	1.02±0.24Bb	1.37±0.66Aa	160.69±83.83Aa	0.64±0.27Ab	0.47±0.19Aa
	Average	51.88±18.98A	8.10±0.55A	12.79±7.38A	1.02±0.23A	1.41±0.74A	170.92±86.22A	0.74±0.33A	0.50±0.17AB

Table S3: Summary of linear mixed-effects models for the effects of environmental conditions on the plant traits across three land uses. *AIC* is Akaike's information criterion. R_m^2 is the variance explained by the fixed effects. R_c^2 is the variance explained by the fixed and random effects. Estimate, R^2, and *P*-value of each fixed term are also exhibited. MAT, mean annual temperature; DOC, dissolved carbon content.

Model <- lmer (Plant traits ~ MAT + Moisture + Salinity + pH + Sulfate + Bulk density + DOC + Soil N + Soil P + Sulfate + (1| Tide)

**Diameter: *AIC* = 124.83; R_m^2 = 0.25; R_c^2 = 0.25**								
	MAT	Moisture	Salinity	pH	Sulfate	DOC	Soil N	Soil P
Estimate	0.092	0.397	-0.152	-0.202	-0.122	0.218	-0.194	0.330
R^2	0.02	0.19	0.13	0.06	0.07	0.12	0.04	0.38
P-value	0.734	0.040	0.088	0.633	0.396	0.049	0.163	0.039
**Height: *AIC* = 115.22; R_m^2 = 0.49; R_c^2 = 0.49**								
	MAT	Moisture	Salinity	pH	Sulfate	DOC	Soil N	Soil P
Estimate	-0.152	0.215	-0.276	-0.370	-0.385	-0.103	0.197	0.297
R^2	0.06	0.04	0.31	0.08	0.31	0.01	0.04	0.14
P-value	0.550	0.235	0.001	0.353	0.006	0.319	0.135	0.049
**Density: *AIC* = 209.19; R_m^2 = 0.46; R_c^2 = 0.50**								
	MAT	Moisture	Salinity	pH	Sulfate	DOC	Soil N	Soil P
Estimate	0.145	-0.697	0.649	2.984	0.560	0.083	-0.224	-0.614
R^2	0.01	0.06	0.39	0.22	0.15	0.01	0.05	0.12
P-value	0.763	0.041	<0.001	<0.001	0.039	0.674	0.362	0.026
**Aboveground biomass: *AIC* = 176.69; R_m^2 = 0.18; R_c^2 = 0.18**								
	MAT	Moisture	Salinity	pH	Sulfate	DOC	Soil N	Soil P
Estimate	0.158	0.195	0.153	1.684	0.018	0.280	-0.208	-0.175
R^2	0.03	0.08	0.13	0.48	0.03	0.16	0.05	0.03
P-value	0.671	0.459	0.210	0.005	0.929	0.066	0.278	0.422
**Specific leaf area: *AIC* = 153.51; R_m^2 = 0.23; R_c^2 = 0.29**								
	MAT	Moisture	Salinity	pH	Sulfate	DOC	Soil N	Soil P
Estimate	-0.748	0.147	0.169	-0.205	0.146	-0.240	0.069	-0.311
R^2	0.22	0.03	0.21	0.07	0.07	0.08	0.02	0.30
P-value	0.026	0.525	0.117	0.698	0.424	0.078	0.681	0.010
**Leaf dry matter content: *AIC* = 162.26; R_m^2 = 0.19; R_c^2 = 0.21**								
	MAT	Moisture	Salinity	pH	Sulfate	DOC	Soil N	Soil P
Estimate	0.506	-0.221	-0.225	0.047	-0.061	0.126	0.002	0.276
R^2	0.15	0.05	0.48	0.03	0.05	0.01	0.02	0.21
P-value	0.145	0.363	0.048	0.932	0.742	0.371	0.991	0.167
**Leaf C content: *AIC* = -102.48; R_m^2 = 0.37; R_c^2 = 0.62**								
	MAT	Moisture	Salinity	pH	Sulfate	DOC	Soil N	Soil P
Estimate	-0.123	-0.134	-0.014	0.618	0.075	-0.058	0.062	0.008
R^2	0.1	0.10	0.07	0.49	0.12	0.05	0.04	0.03
P-value	0.074	0.004	0.518	<0.001	0.054	0.038	0.076	0.835

Table S3: Summary of linear mixed-effects models for the effects of environmental conditions on the plant traits across three land uses. *AIC* is Akaike's information criterion. R_m^2 is the variance explained by the fixed effects. R_c^2 is the variance explained by the fixed and random effects. Estimate, R^2, and *P*-value of each fixed term are also exhibited. MAT, mean annual temperature; DOC, dissolved carbon content.

Model <- lmer (Plant traits ~ MAT + Moisture + Salinity + pH + Sulfate + Bulk density + DOC + Soil N + Soil P + Sulfate + (1| Tide)

**Leaf N content:** ***AIC*** **= 62.95;** R_m^2 **= 0.27;** R_c^2 **= 0.76**								
	MAT	Moisture	Salinity	pH	Sulfate	DOC	Soil N	Soil P
Estimate	-1.180	-0.379	-0.014	0.542	0.322	-0.186	0.295	0.044
R^2	0.49	0.13	0.03	0.03	0.09	0.06	0.13	0.03
P-value	<0.001	0.004	0.817	0.078	0.003	0.016	0.002	0.669
**Leaf P content:** ***AIC*** **= 40.20;** R_m^2 **= 0.52;** R_c^2 **= 0.55**								
	MAT	Moisture	Salinity	pH	Sulfate	DOC	Soil N	Soil P
Estimate	0.284	0.365	-0.029	0.671	0.065	0.086	0.061	-0.073
R^2	0.13	0.30	0.04	0.07	0.17	0.09	0.14	0.05
P-value	0.086	0.002	0.583	0.012	0.473	0.202	0.465	0.431
**Leaf C:N ratio:** ***AIC*** **= -24.97;** R_m^2 **= 0.22;** R_c^2 **= 0.95**								
	MAT	Moisture	Salinity	pH	Sulfate	DOC	Soil N	Soil P
Estimate	0.970	0.023	0.046	0.212	-0.099	0.066	-0.287	0.019
R^2	0.59	<0.01	0.02	0.01	0.09	0.08	0.22	<0.01
P-value	<0.001	0.794	0.308	0.512	0.204	0.376	<0.001	0.865
**Leaf C:P ratio:** ***AIC*** **= -20.97;** R_m^2 **= 0.26;** R_c^2 **= 0.95**								
	MAT	Moisture	Salinity	pH	Sulfate	DOC	Soil N	Soil P
Estimate	-0.593	-0.237	-0.007	-0.485	-0.001	0.010	-0.065	0.180
R^2	0.59	0.26	0.03	<0.01	0.13	<0.01	0.02	<0.01
P-value	0.040	0.007	0.875	0.172	0.995	0.901	0.352	0.142
**Leaf N:P ratio:** ***AIC*** **= 31.13;** R_m^2 **= 0.32;** R_c^2 **= 0.96**								
	MAT	Moisture	Salinity	pH	Sulfate	DOC	Soil N	Soil P
Estimate	-1.547	-0.225	-0.064	-0.808	0.063	-0.038	0.237	0.185
R^2	0.84	0.04	0.03	0.02	<0.01	0.01	0.05	<0.01
P-value	<0.001	0.062	0.293	0.096	0.562	0.720	0.015	0.265

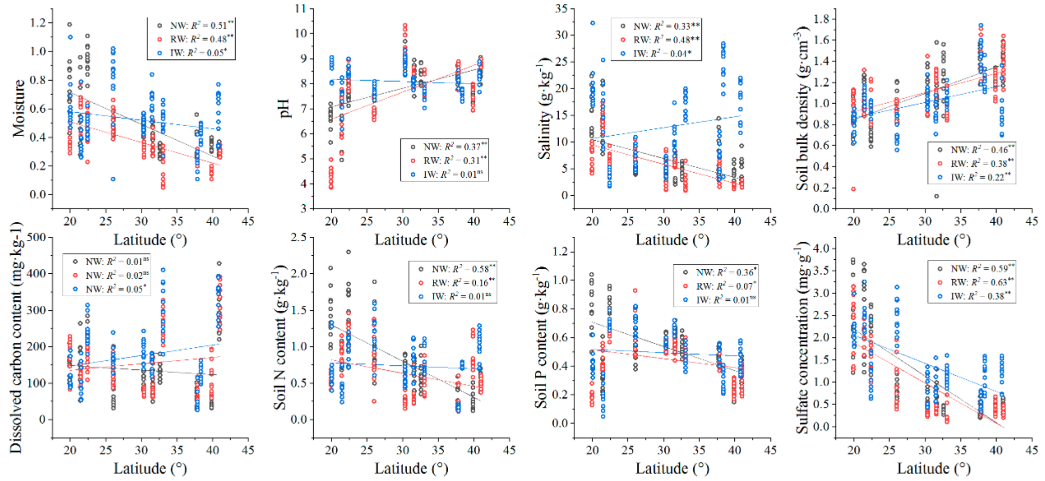

Figure S1: Relationships between soil properties and latitude. * $P < 0.05$; ** $P < 0.01$; ns, $P > 0.05$. IW, invasive wetlands. NW, natural wetlands. RW, reclaimed wetlands.

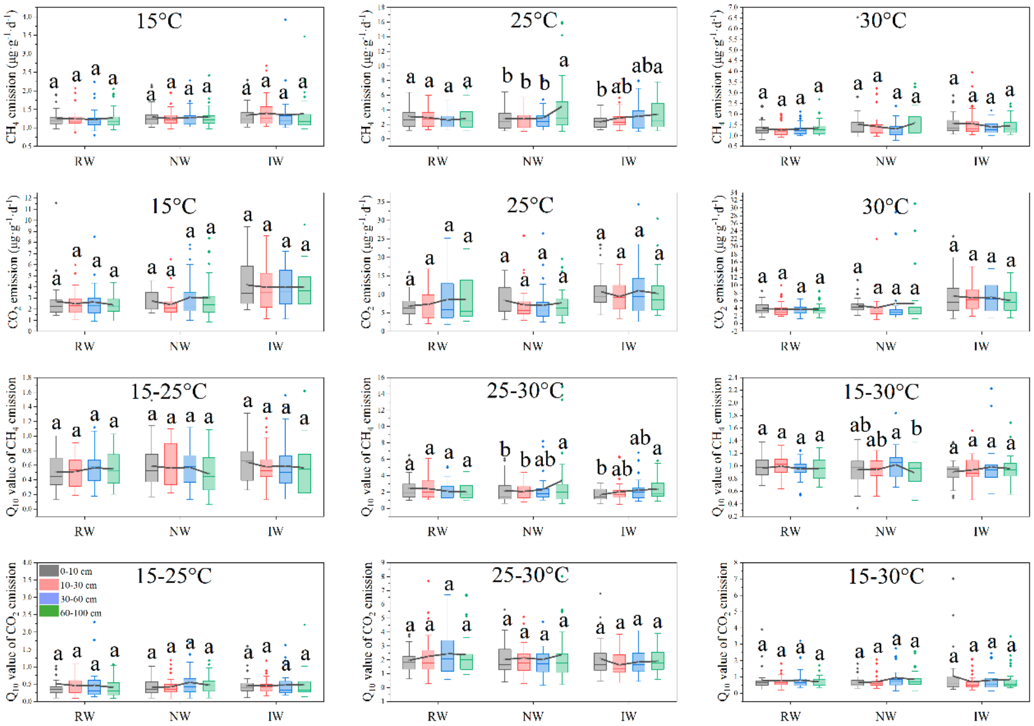

Figure S2: Distribution of CH_4 and CO_2 emissions and their Q_{10} values in different land uses among different soil depths and incubation temperatures. Different letters indicate a significant difference among different land uses ($P < 0.05$). IW, invasive wetlands. NW, natural wetlands. RW, reclaimed wetlands.

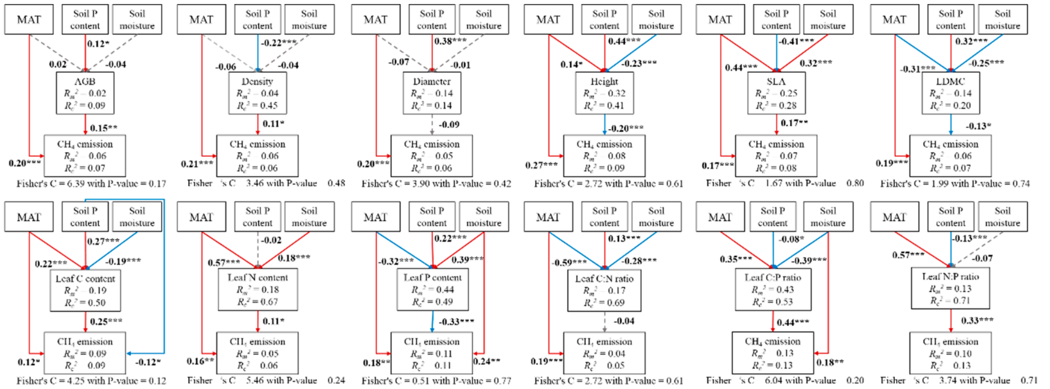

Figure S3: Structure equation modeling (SEM) results of influential pathways on CH_4 emission. MAT represents PC1 from PCA conducted with mean annual temperature and precipitation. Soil moisture and soil P content represent PC1 and PC2 from PCA, respectively, which was conducted with soil moisture, pH, salinity, bulk density, sulfate concentration, dissolved carbon content, and N and P contents. Goodness-of-fit statistics for the models are shown below the models. R_m^2 represents marginal R^2. R_c^2 represents conditional R^2. Gray arrows with dashed lines represent non-significant pathways. Blue or red solid lines represent significant negative or positive pathways. *$P < 0.05$; **$P < 0.01$; ***$P < 0.001$.

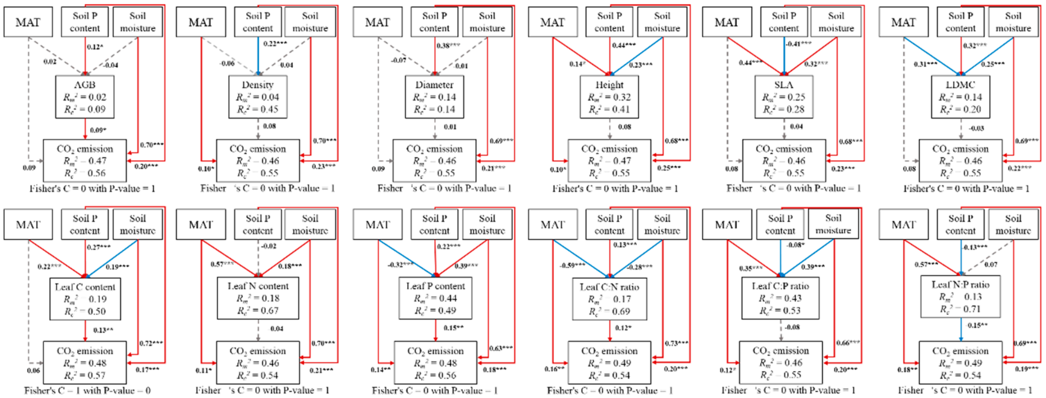

Figure S4: Structure equation modeling (SEM) results of influential pathways on CO_2 emission. MAT represents PC1 from PCA conducted with mean annual temperature and precipitation. Soil moisture and soil P content represent PC1 and PC2 from PCA, respectively, which was conducted with soil moisture, pH, salinity, bulk density, sulfate concentration, dissolved carbon content, and N and P contents. Goodness-of-fit statistics for the models are shown below the models. R_m^2 represents marginal R^2. R_c^2 represents conditional R^2. Gray arrows with dashed lines represent non-significant pathways. Blue or red solid lines represent significant negative or positive pathways. *$P < 0.05$; **$P < 0.01$; ***$P < 0.001$.

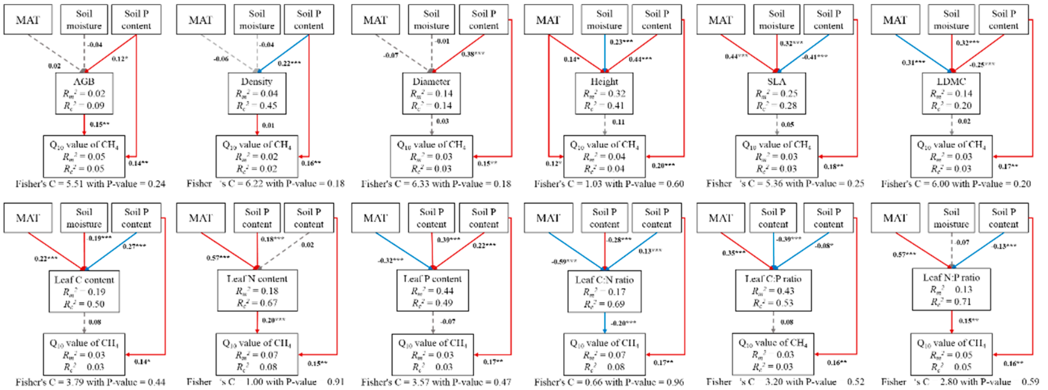

Figure S5: Structure equation modeling (SEM) results of influential pathways on the Q_{10} value of CH_4 emission. MAT represents PC1 from PCA conducted with mean annual temperature and precipitation. Soil moisture and soil P content represent PC1 and PC2 from PCA, respectively, which was conducted with soil moisture, pH, salinity, bulk density, sulfate concentration, dissolved carbon content, and N and P contents. Goodness-of-fit statistics for the models are shown below the models. R_m^2 represents marginal R^2. R_c^2 represents conditional R^2. Gray arrows with dashed lines represent non-significant pathways. Blue or red solid lines represent significant negative or positive pathways. *$P < 0.05$; **$P < 0.01$; ***$P < 0.001$.

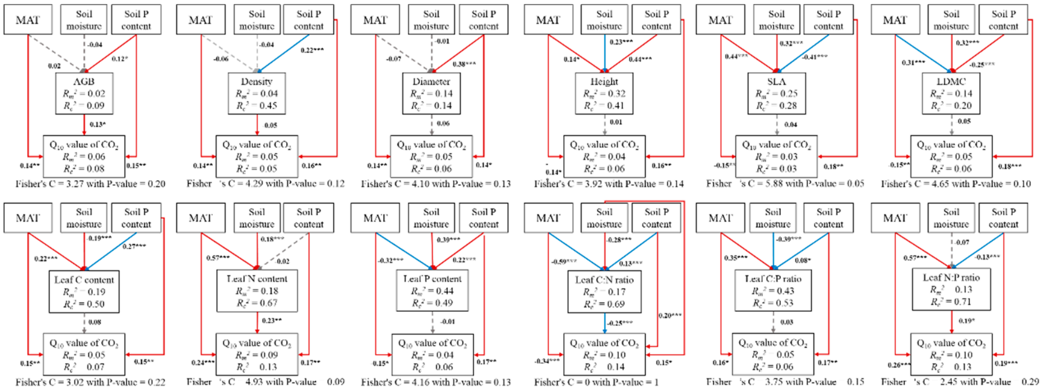

Figure S6: Structure equation modeling (SEM) results of influential pathways on the Q_{10} value of CO_2 emission. MAT represents PC1 from PCA conducted with mean annual temperature and precipitation. Soil moisture and soil P content represent PC1 and PC2 from PCA, respectively, which was conducted with soil moisture, pH, salinity, bulk density, sulfate concentration, dissolved carbon content, and N and P contents. Goodness-of-fit statistics for the models are shown below the models. R_m^2 represents marginal R^2. R_c^2 represents conditional R^2. Gray arrows with dashed lines represent non-significant pathways. Blue or red solid lines represent significant negative or positive pathways. *$P < 0.05$; **$P < 0.01$; ***$P < 0.001$.

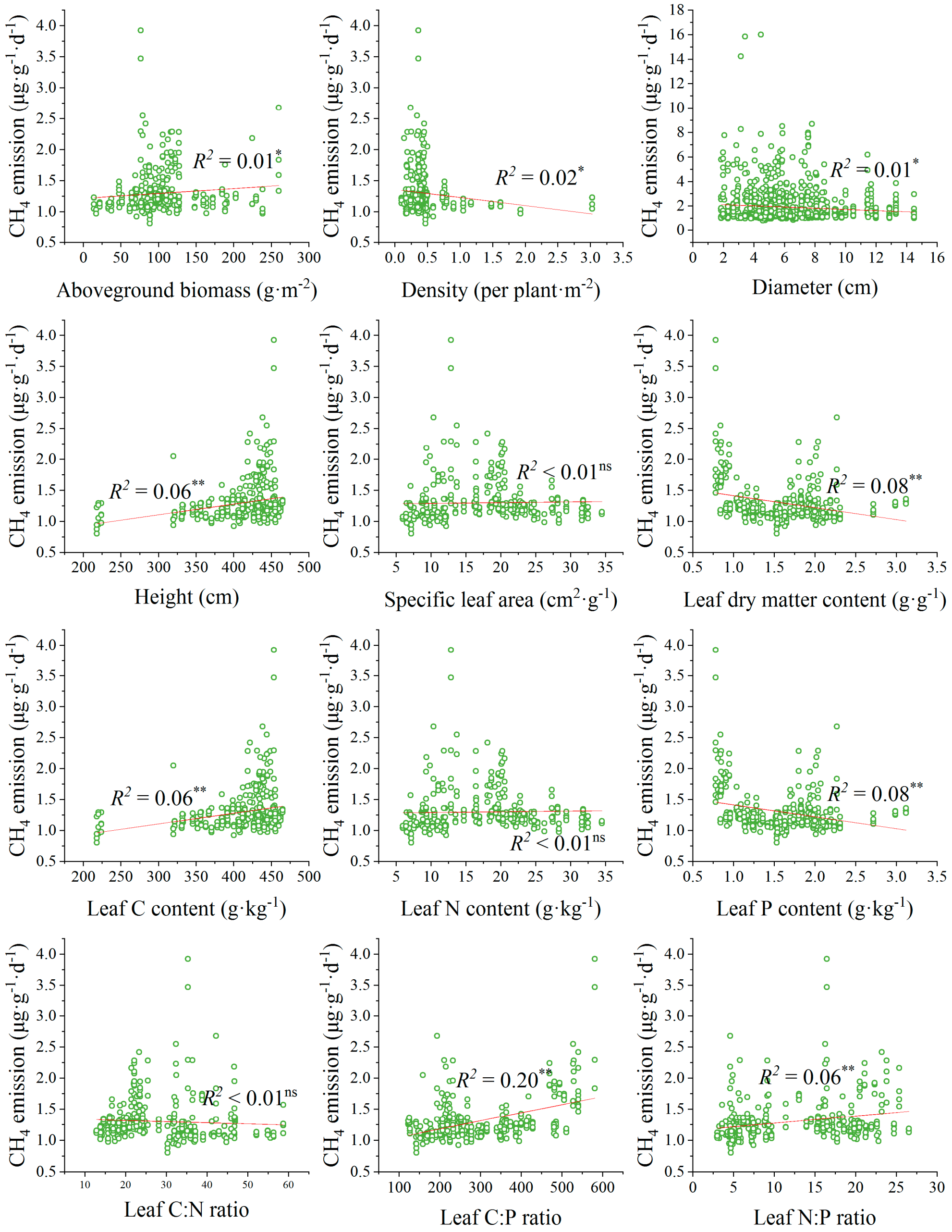

Figure. S7: Linear relationships between plants and CH_4 emission.

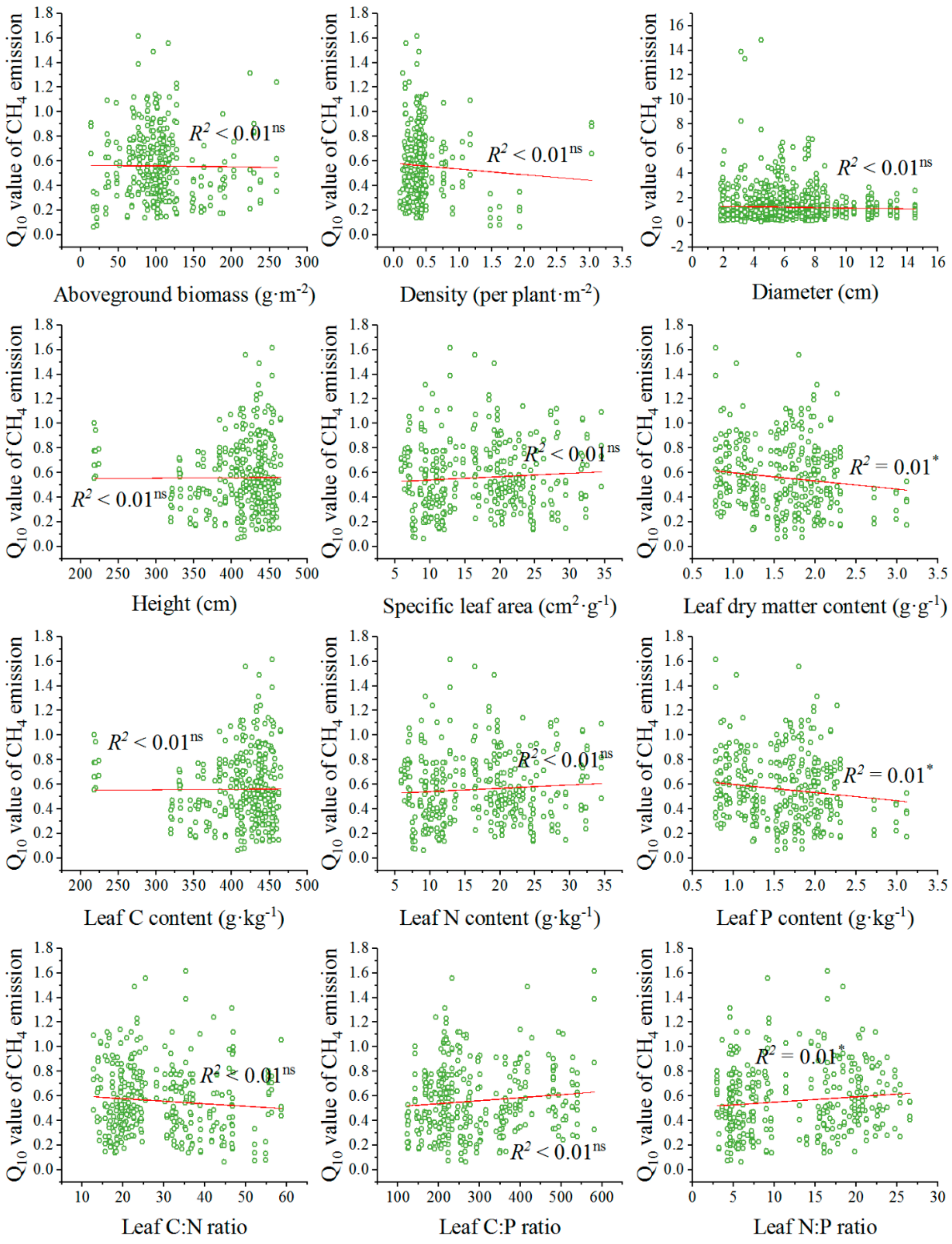

Figure S8: Linear relationships between plants and Q_{10} value of CH_4 emission.

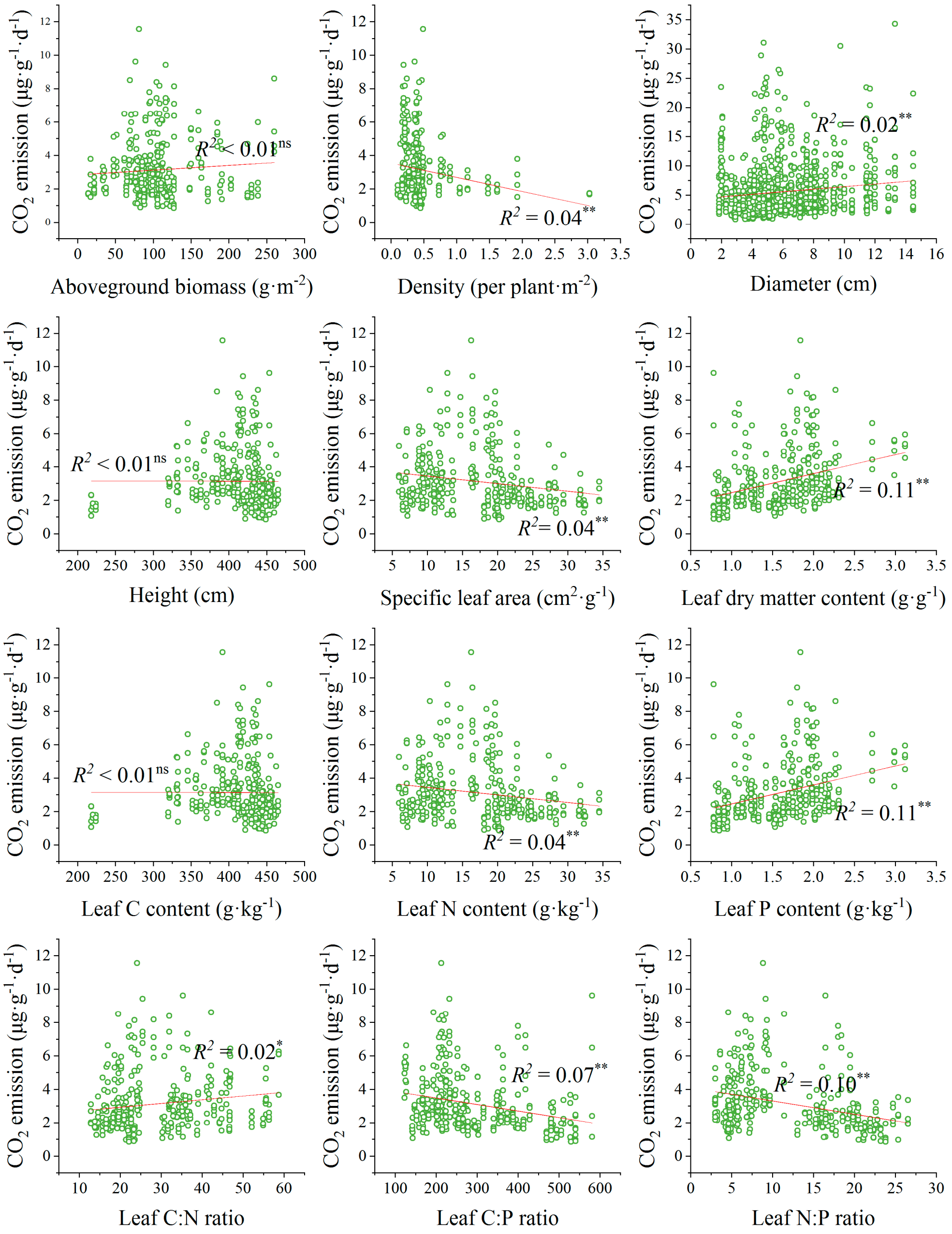

Figure S9: Linear relationships between plants and CO_2 emission.

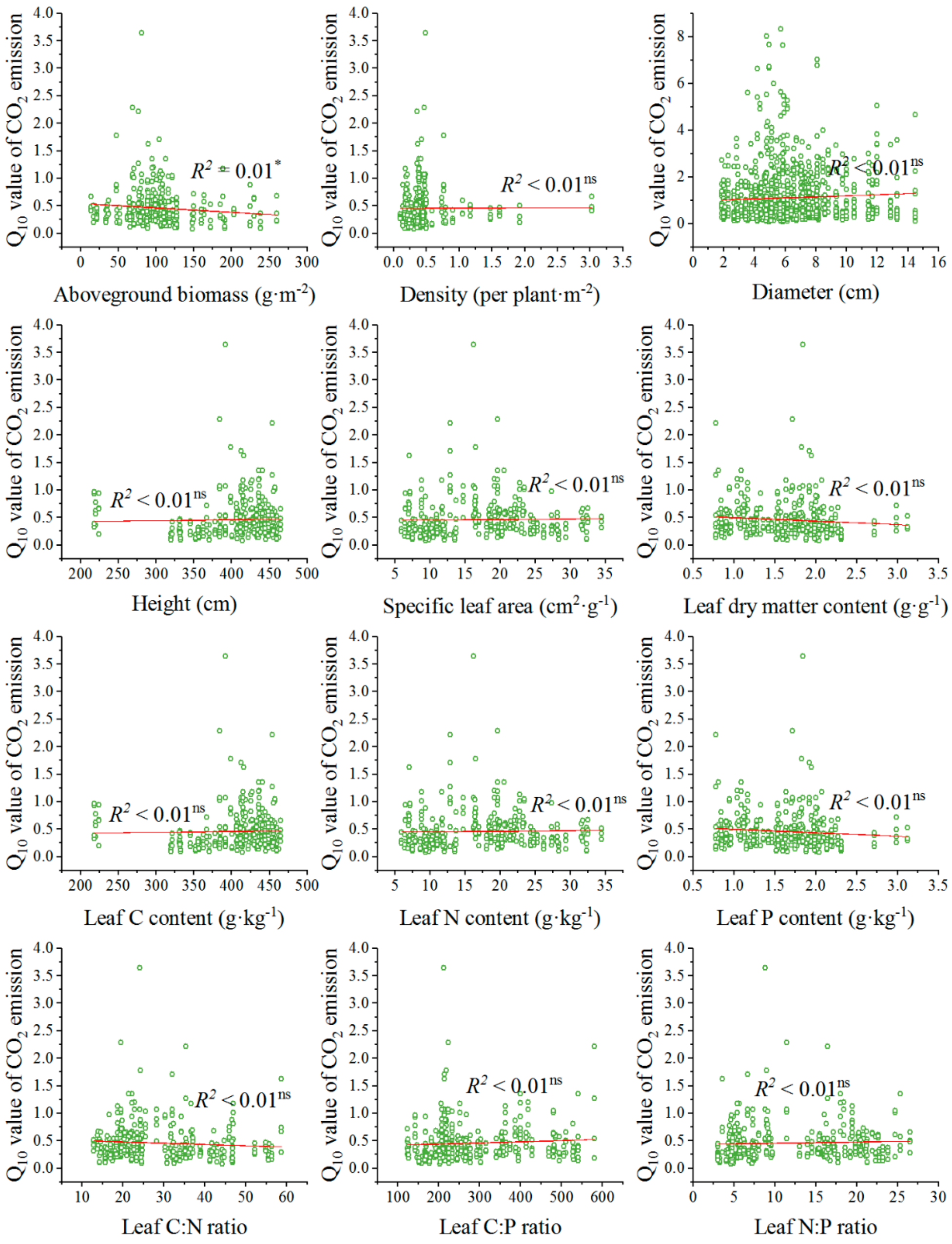

Figure S10: Linear relationships between plants and Q_{10} value of CO_2 emission.
